# Isolation and identification of a novel phage targeting clinical multidrug-resistant *Corynebacterium striatum* isolates

**DOI:** 10.3389/fcimb.2024.1361045

**Published:** 2024-03-20

**Authors:** Jiao Wang, Meng Zhang, Jiao Pei, Wei Yi, Li Fan, Chunhua Wang, Xiao Xiao

**Affiliations:** ^1^ Department of Pathogen Biology, School of Basic Medicine, Hubei University of Arts and Science, Xiangyang, China; ^2^ Xiangyang Central Hospital, Affiliated Hospital of Hubei University of Arts and Science, Xiangyang, China; ^3^ Department of Clinical Laboratory, Xiangyang No.1 People’s Hospital, Hubei University of Medicine, Xiangyang, China; ^4^ Department of Pathogen Biology, School of Basic Medicine, Hubei University of Medicine, Shiyan, China

**Keywords:** multidrug-resistance *Corynebacterium striatum* (MDR-*C. striatum*), *Caudoviricetes*, temperate phage, genome analysis, site-specific recombination element

## Abstract

**Introduction:**

Over the past decade, Corynebacterium striatum (C. striatum), an emerging multidrug-resistant (MDR) pathogen, has significantly challenged healthcare settings, especially those involving individuals with weakened immune systems. The rise of these superbugs necessitates innovative solutions.

**Methods:**

This study aimed to isolate and characterize bacteriophages targeting MDR-C. striatum. Utilizing 54 MDR-C. striatum isolates from a local hospital as target strains, samples were collected from restroom puddles for phage screening. Dot Plaque and Double-layer plate Assays were employed for screening.

**Results:**

A novel temperate bacteriophage, named CSP1, was identified through a series of procedures, including purification, genome extraction, sequencing, and one-step growth curves. CSP1 possesses a 39,752 base pair circular double-stranded DNA genome with HK97-like structural proteins and potential for site-specific recombination. It represents a new species within the unclassified Caudoviricetes class, as supported by transmission electron microscopy, genomic evolutionary analysis, and collinearity studies. Notably, CSP1 infected and lysed 21 clinical MDR-C. striatum isolates, demonstrating a wide host range. The phage remained stable in conditions ranging from -40 to 55°C, pH 4 to 12, and in 0.9% NaCl buffer, showing no cytotoxicity.

**Discussion:**

The identification of CSP1 as the first phage targeting clinical C. striatum strains opens new possibilities in bacteriophage therapy research, and the development of diagnostic and therapeutic tools against pathogenic bacteria.

## 1 Introduction

*Corynebacterium striatum* (*C. striatum*), a member of the *Corynebacterium* genus, is a Gram-positive bacterium naturally present in the environment, commonly colonizing mucosal sites such as human skin and nasopharynx. Over the past decade, *C. striatum* has increasingly been identified as a significant opportunistic pathogen in both community-acquired and hospital-acquired infections, with a rising frequency in clinical sample isolations (McMullen et al., 2017; Lee et al., 2022). Identified as a pathogen since 1980 (Bowstead and Santiago, 1980), *C. striatum* has gained prominence due to its biofilm-forming ability, enhancing survival within hosts and medical environments and bolstering resistance to antibiotics and immune attacks (Alibi et al., 2021). It causes various conditions in patients with chronic diseases and compromised immunity, including bacteremia, septicemia, septic arthritis, meningitis, and respiratory infections (Leyton et al., 2021; Silva-Santana et al., 2021). As a notable emerging pathogen, *C. striatum* exhibits substantial variability and adaptability (Renom et al., 2014), prevalently found in clinical sputum and secretion samples, especially during summer, and shows a tendency for rapid spread and evolution in different clinical departments, leading to multidrug resistance (Wang et al., 2022). Currently, vancomycin remains the primary treatment for *C. striatum* infections, with linezolid, tigecycline, or daptomycin as alternatives for severe cases (Milosavljevic et al., 2021). The COVID-19 pandemic may have exacerbated the global antibiotic resistance crisis (Yam, 2020), with predictions that by 2030, vancomycin might be the only effective antibiotic remaining (Orosz et al., 2022).

The extensive use of antibiotics has led to multiple issues, such as increased antibiotic-resistant bacteria, immune suppression, drug residues in animal products, and environmental pollution (Liang et al., 2023). Notably, antibiotic resistance has become a significant global concern. The Chinese government has acknowledged the seriousness of bacterial drug resistance, incorporating it into the agenda of the G20 Hangzhou Summit to address the growth of bacterial resistance (Liang et al., 2023). Phages, as natural predators of bacteria, are the most diverse and abundant entities on Earth, posing no adverse effects on normal bacterial communities (Dion et al., 2020). Amid the escalating challenge of global multi-drug and pan-drug resistant bacterial pathogens, phages and their derivative peptidoglycan hydrolases are considered potent supplements to antibiotics (Moelling et al., 2018; Ramesh et al., 2022; Taslem Mourosi et al., 2022), emerging as promising antimicrobial tools (Domingo-Calap et al., 2016). Encouragingly, studies show that combining phages with antibiotics—resulting in a strong synergistic effect—enhances treatment outcomes for specific infections beyond what either can achieve independently. This synergy likely arises from improved bacterial killing, better biofilm penetration, and a decrease in the selection of resistant clones. Yet, this phenomenon is not universal, and its underlying mechanisms require further clarification (Pirnay et al., 2022). Phages have been used in treating various severe infections, including *Staphylococcus aureus* bacteremia, typhoid, and osteomyelitis (d’Herelle, 1931), combating intracellular pathogens (Goswami et al., 2021), controlling bacterial plant diseases in agriculture, such as apple blossom infection caused by *Erwinia amylovora* (Schnabel and Jones, 2001; Halawa, 2023), and mitigating food contamination with Enterobacter, Salmonella, Listeria, and Vibrio (Endersen et al., 2014). Despite the absence of global standards for phage application, the rapid development in phage therapy, coupled with China’s challenge in reducing antibiotic dependence, has prompted the isolation and screening of clinical resistant bacterial phages as a last line of defense against antibiotic failure (Monteiro et al., 2019).

Presently, research on phages targeting clinically resistant *Corynebacterium striatum* isolates remain scarce. IME1320_01 is the only reported inducible phage identified to date. Its genome, present as a prophage on the *Corynebacterium striatum* strain 1320 chromosome, produces phage particles when induced by mitomycin C (Zhao et al., 2019). However, its infectivity has not been proven by double-layer agar plate assays, possibly due to bacterial immune systems like the CRISPR-Cas and restriction-modification systems, prophage-encoded defense mechanisms (e.g., toxin-antitoxin components), or low phage quantities (Hampton et al., 2020). This study introduces a newly isolated phage from a hospital environment, named *Corynebacterium striatum* phage CSP1, belonging to the *Caudoviricetes* class. Despite carrying lysogenic gene modules for integration, such as site-specific recombination functional elements (integrase and recombination sites) (Wang et al., 2018), CSP1 targets up to 21 multidrug-resistant *C. striatum* strains from various clinical department samples (Wang et al., 2022). This discovery provides a foundation for the utilization of *C. striatum* phages as diagnostic and therapeutic vectors, targeting pathogenic bacteria in medical research.

## 2 Materials and methods

### 2.1 Bacterial strain, phage isolation, and enrichment

The bacterial strains used in this study were clinical isolates of multi-drug resistant *Corynebacterium striatum*, totaling 54 strains (labeled as *Cs*1 to *Cs*54) (Wang et al., 2022). Eight samples of wastewater or sludge were collected from the Xiangyang Central Hospital, affiliated with the Hubei University of Arts and Science in Xiangyang, Hubei Province, China. For liquid samples, a low-speed centrifugation at 4°C was first performed, followed by filtration through a 0.22 μm filter to collect the supernatant. For solid (sludge) samples, sterile PBS solution was added, stirred for 1 hour, then centrifuged, and processed similarly to the liquid samples to obtain the supernatant.

To enhance the concentration of phages in the samples, 20 mL of the supernatant from each sample was added to 100 mL of logarithmic-phase *Corynebacterium striatum* culture. The mixture was then incubated at 37°C and 200 r/min for 10 hours. Subsequently, the mixtures were centrifuged at 12000 r/min for 5 minutes to collect and filter the supernatant, repeating this process three times to enrich the environmental phage samples.

The presence of phages in the enriched samples was initially screened using the Plaque dot assay (Powell et al., 1986; Guan et al., 2022). 50 μL of the host bacteria (*Cs*1 to *Cs*54) was mixed with 4 mL of semi-solid medium and poured onto double-layer plates. After solidification, 2 μL of the filtered liquid from the eight enriched samples (marked as ①②③④⑤⑥⑦⑧) was spotted on the plates and incubated overnight at 30°C. The formation of plaques was observed the following day.

### 2.2 Phage purification and concentration

Following the initial screening of phages in environmental samples using the dot plaque assay, the phages were isolated and purified. The enriched supernatant was first filtered through a 0.22 µm filter to obtain a crude phage solution, which was then serially diluted in ten-fold increments from 10^-1^ to 10^-5^. One hundred µL of each diluted phage solution was mixed with 50 µL of logarithmic-phase *Cs*11 bacterial culture and incubated at room temperature for 10 minutes. This mixture was then added to 4 mL of semi-solid medium (0.65% agar) and poured onto LB agar plates, incubated overnight at 37°C using the double-layer plate method (Bai et al., 2022). Independent and clear plaques on the double-layer plates were picked with a sterile toothpick into 1 mL of sterile liquid medium, vortexed, and then serially diluted to 10^-6^. One hundred µL of the original and each diluted phage solution was mixed with 50 µL of *Cs*11 bacterial culture, poured onto double-layer plates, and incubated overnight at 37°C, with plaque morphology observed. This step was repeated at least three times until plaques of consistent appearance and size were obtained on the same double-layer plate, indicating phage purification.

For subsequent characterization experiments, the purified phages were amplified and concentrated. Twenty mL of purified phage solution was added to 100 mL of logarithmic-phase *Cs*11 bacterial culture and incubated overnight at 37°C with 200 r/min shaking, followed by centrifugation at 12000 r/min for 5 minutes to collect and filter the supernatant. This process was repeated three times to amplify the phages. The phages were then concentrated using an ultrafiltration method to increase the phage titer. Specifically, 20 mL of phage filtrate was transferred to a 50 mL ultrafiltration centrifuge tube (15 mL, 10 KDa, Millipore UFC901096) and centrifuged at 3000 rpm for 20 minutes. This process was repeated until approximately 600 mL of phage original solution was concentrated by about 30 times to obtain the concentrated phage solution.

### 2.3 Morphological observation of bacteriophage

A 10 µL aliquot of the concentrated bacteriophage storage solution was dropped onto a clean parafilm. Subsequently, a 300-mesh carbon-coated nickel grid (EMCN BZ11032a, China) was placed onto the droplet and left to stand for 2 minutes before the grid was removed. Excess liquid was then absorbed from the side of the grid using filter paper. Next, 10 µL of 2% phosphotungstic acid was dropped onto the parafilm, and the grid with the phage liquid was placed onto this droplet, incubated for 1 minute, then lifted, and excess staining fluid was absorbed from the side with filter paper. The grid was allowed to air dry, face up, on filter paper at room temperature for 15-20 minutes. The morphology of the bacteriophage was observed and photographed using a transmission electron microscope under an 80 KV microscope software setup.

### 2.4 Phage host range

The host range of the phage was determined based on 54 MDR-*C. striatum* (Wang et al., 2022), along with 7 strains of *Corynebacterium pseudodiphtheriticum* (isolated from the secretion sample of a patient with sinusitis in an Otorhinolaryngology department), *Escherichia coli* (DH5α, DHM1, BL21), *Staphylococcus aureus*, *Acinetobacter baumannii*, and *Enterococcus faecalis*. The Plaque dot assay method was employed, where 2 μL of phage solution at various dilution gradients (10^0^, 10^-1^, 10^-2^, 10^-3^, 10^-4^) were applied onto double-layer agar plates of different test-sensitive bacteria. After incubation at 37°C for 12 hours, the formation of gradient plaques on the bacterial lawns of the tested strains was observed. Detailed information about the clinical departments, resistance profiles, and *16S rRNA* gene sequencing analysis of the MDR-*C. striatum* isolates can be found in our previously published paper (Wang et al., 2022). The phylogenetic tree of phage-sensitive bacteria was constructed using the MEGA 11 software package, as described in our earlier work (Wang et al., 2022).

### 2.5 Phage DNA extraction and enzyme digestion

Phage DNA was extracted using the EasyPure® Viral DNA/RNA Kit (ER201-01, Transgen Biotech Co., LTD) following the manufacturer’s protocol. The extracted genomic DNA was then subjected to electrophoresis on a 0.7% agarose gel, and samples were quantified and qualified using a NanoDrop™ One Spectrophotometer (Thermo Scientific™) for subsequent experiments. The extracted phage genomes were temporarily stored at -40°C.

To identify the type of phage nucleic acid, 8 μL of phage genome (100 ng/μL) was taken and added to different enzyme digestion systems (total volume 20 μL), including 0.5 μL DNase I, 0.5 μL RNase A, 0.2 μL (0.1 μg/μL) Mung Bean Nuclease, and 0.5 μL Exonuclease III (Exo III). Except for the Mung Bean Nuclease digestion system, which was incubated at 30°C for 30 minutes, all other enzymes were incubated at 37°C for 1 hour. The enzyme digestion products were mixed with 6 x DNA Loading buffer in a ratio of 5:1 and then loaded onto a 0.7% agarose gel for analysis. The analysis determined the phage genome type, whether double or single-stranded, and whether linear or circular. Fresh linear double-stranded fragments (1800 bp, 120 ng/μL) and circular plasmids (6000 bp, 91 ng/μL) were used as controls.

### 2.6 Genomic sequencing, annotation, and bioinformatics analysis

Three samples of the extracted phage genomes, corresponding to environmental samples numbered ①, ③, and ⑦, were sent to AuGCT Biotechnology Co., Ltd. (Wuhan, China) for sequencing. Initially, the phage genomes were randomly fragmented into approximately 500 bp segments. The required DNA fragments were collected, and specific adapters were selected using the NEB standard library preparation kit. The library fragment size was assessed using the Agilent 2100 and the library molar concentration was determined by qPCR. Upon successful library verification, sequencing was performed using the Illumina NovaSeq with PE 2*150, constituting ultra-deep NGS. The project utilized SPAdes (ver3.15.4) software for sequence assembly, with the corrected final result serving as the standard sequence. The mapping rate of the cleaning data was calculated. Annotation involved predicting CDS regions using prokka (Seemann, 2014) and annotating CDS with the egNOG database using the HMM algorithm. After BLAST (Basic Local Alignment Search Tool) comparison, the PHAge Search Tool (PHAST) (Zhou et al., 2011) was used for online phage component prediction. Additionally, new annotations were made based on the current NCBI nr database, followed by manual verification and correction based on the KEGG and NCBI databases. Protein annotations were used if the full-length coverage was over 80% and similarity was not less than 60% (preferably over 80%), otherwise labeled as a new protein (hypothetical protein). For the phylogenetic and taxonomic analysis of phage genomes, VCTOR (Meier-Kolthoff and Göker, 2017) online analysis was used (https://ggdc.dsmz.de/victor.php). Cluster analysis and collinearity studies of multiple genomic sequences were visualized using Easyfig (Sullivan et al., 2011). Functional similarity prediction and determination between two proteins were conducted through pairwise similarity calculations of protein sequences using the EMBOSS Needle tool (http://www.ebi.ac.uk/Tools/psa/embossneedle/). Prediction of unknown protein functions was carried out using the HHpred server (https://toolkit.tuebingen.mpg.de/tools/hhpred) (Söding et al., 2005), based on remote protein homology detection and structure prediction.

### 2.7 Phage optimal multiplicity of infection and one-step growth curve determination

To ascertain the optimal multiplicity of infection (MOI) for phage isolate CSP1, this study mixed varying ratios of phage CSP1 with *Cs*11 strain. Based on the titration of phage CSP1 and the bacterial count in the culture (approximately 2.5×10^8^ CFU/mL at OD_600 nm_ of 0.4), MOI ratios were set at 0.001, 0.01, 0.1, 1, 10, and 100. A mixture of 1mL of phage CSP1 and 1mL of bacterial culture was inoculated into 100 mL of fresh LB liquid medium and incubated at 37°C for 8 hours. Subsequently, the culture was centrifuged (8000 rpm/min for 10 minutes) and the supernatant filtered through a 0.22 μm filter to remove bacteria, followed by determining the phage CSP1 titer (PFU/mL) (calculated as the number of CSP1 plaques on double-layer agar plates × dilution factor × 10). The highest phage release under different MOI ratios was recorded to determine the optimal MOI (Fang et al., 2023). The assay was performed in triplicate.

Following the standard method for phage one-step growth curve determination (Gašić et al., 2022), phage CSP1 and *Cs*11 strain were inoculated into LB liquid medium at the optimal MOI and incubated at 37°C for 30 minutes. Unadsorbed phages were removed by centrifugation at 4°C at 13000 rpm for 20 minutes, discarding the supernatant. The pellet was washed twice with fresh LB liquid medium, resuspended, and further incubated on a 37°C shaker. Phage titers were measured every 10 minutes for a duration of 100 minutes, totaling 11 samples (including a zero-time point sample), to determine the burst period of phage CSP1 (each set of experiments was repeated thrice).

### 2.8 Phage stability analysis and bacteriolytic activity *in vitro*

To evaluate the impact of external physicochemical factors on the stability of phage CSP1, factors such as ultraviolet (UV) light, temperature, medium pH, NaCl concentration, and disinfectants were investigated. In the UV sensitivity assay, 1mL of phage CSP1 solution was poured into a sterile petri dish (uncovered) and placed horizontally under a 30 W UV lamp inside a clean bench with internal dimensions of 1340 mm × 540 mm × 545 mm. The phage CSP1 titer was measured following exposure to UV light for durations of 0, 2, 4, 6, 7, 8, 10, and 15 minutes. For the temperature stability assay, 1mL of phage CSP1 solution was placed in a 1.5mL Eppendorf tube and subject-ed to water baths set at -40°C, -20°C, 4°C, 25°C, 37°C, 42°C, 55°C, and 60°C. After incubation for 1 hour, the effect of these temperature treatments on phage titer was assessed (each set of experiments was repeated thrice).

To evaluate the effect of pH on phage stability, the pH of the liquid medium was adjusted to values ranging from 2.0 to 12.0 using 1M sodium hydroxide and hydrochloric acid solutions (pH remained stable after sterilization). A volume of 50 μL of phage CSP1 was then added to 5 mL of the medium at each pH value, followed by incubation at 37°C for 1 hour, with titers determined using the double-layer agar plate method. Additionally, to assess the effects of NaCl concentration and disinfectants on the phage, the original phage CSP1 solution was diluted to 1% and added to solutions of 0.9% NaCl and 2% glutaraldehyde. After incubation at room temperature for 1 hour, phage titers were determined (experiments were repeated at least thrice).

To determine the lytic activity of bacteriophage CSP1, the *Cs*11 strain (in its logarithmic growth phase, OD_600 nm_ = 0.22; bacterial count Log10 = 8.32 ± 0.13) was inoculated at 1% (v/v) into LB broth (150 mL) and infected with phage CSP1 at a multiplicity of infection (MOI) of 100, 10, 1, 0.1, 0.01, or 0.001 at 37°C under aerobic conditions (150 rpm/min). Culture samples were collected at various time intervals (15 min, 45 min, 8.5 h, 10.5 h, 11.5 h, 12.5 h, 13.5 h, and 14.5 h) for 14.5 hours, and bacterial growth was assessed based on the optical density at 600 nm (OD_600 nm_). Additionally, photographic observations of the liquid cultures were made at key time points to qualitatively assess the state of the cultures. All experiments were conducted in triplicate.

### 2.9 Preliminary assessment of phage cell safety

To investigate the safety of phage isolate CSP1 on human cells, we utilized the Cell Counting Kit-8 (CCK-8) (GLPBIOGK10001) to assess the cytotoxic effects of CSP1 on human embryonic kidney cells 293 (HEK293 T) and lung adenocarcinoma cells (A549) *in vitro*. HEK293T and A549 cells were seeded in a 96-well plate at a density of 5,000 cells/well in 100 μL. Each well received 10 μL of the bacteriophage CSP1 at initial concentrations of 3×10^12^ PFU/mL, followed by a series of tenfold dilutions, resulting in phage concentrations of 3×10^10^, 3×10^9^, 3×10^8^, and 3×10^7^ PFU/well. For the control setup, wells 1 and 2 each held 100 μL of cell suspension; well 1 was augmented with 10 μL of LB medium, while well 2 received 10 μL of standard cell culture medium. Following treatment, the plates were incubated in a cell culture incubator for 12 or 24 hours, after which CCK-8 was added to each well except for three replicate wells per experimental group, which did not receive CCK-8 to allow for the determination of phage titer in the phage-cell co-culture. The incubation was continued at 37°C for an additional hour. The optical density at 420 nm was measured using the Molecular Devices SpectraMax®i3 plate reader, and data were analyzed in triplicate for each condition. Additionally, the titer of bacteriophage CSP1 without CCK-8 in the experimental groups was determined.

### 2.10 Statistical analysis

All data were analyzed using GraphPad Prism 8.0.2 and expressed as means and standard deviation values. One-way analysis of variance (ANOVA) was used to compare multiple groups. Statistical significance was set at p<0.05.

## 3 Results

### 3.1 Phage plaque and morphology

**Figure 1:**
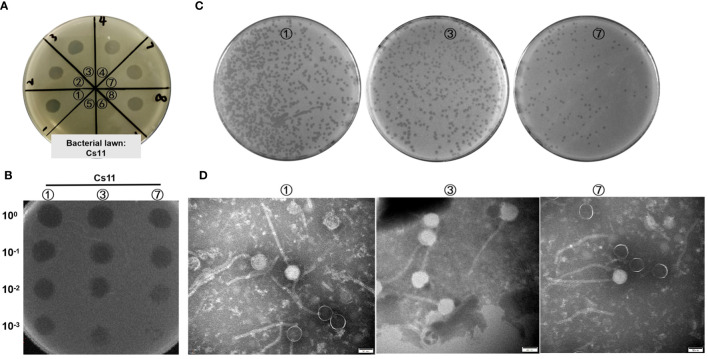
Visualization of phage samples through plaque formation and Transmission Electron Microscopy. **(A)** Preliminary screening for phages in environmental samples utilizing the dot plaque method (*Cs11* used as the sensitive bacterial strain; eight environmental samples were analyzed, labeled as ① to ⑧). **(B)** Identification of phages in three environmental samples (①, ③, and ⑦) using the dilution Dot plaque method. **(C)** Morphological presentation of phage plaques on double-layer agar plates derived from the purified phage samples. **(D)** Morphological characterization of phages using transmission electron microscopy.

In this study, the dot plaque method (Powell et al., 1986; Guan et al., 2022) was initially employed for preliminary screening of environmental samples. On double-layer agar plates using *C. striatum* 11 (*Cs*11) as the susceptible bacterial strain, transparent plaques were observed in the enriched cultures from 8 environmental samples (as shown in **Figure 1A**), suggesting the potential presence of bacteriophages. To eliminate interference from bacteriocins or other antibacterial substances, a ten-fold serial dilution in the phage dot plaque assay was further conducted. The results revealed that only 3 samples (labeled ①, ③, and ⑦) conclusively contained bacterio-phage particles, while the other 5 samples failed to form plaques upon dilution (**Figure 1B**). Subsequently, through the double-layer agar method (Bai et al., 2022) and six rounds of purification, 3 distinct bacteriophage strains were isolated from these samples, designated as phage ①/③/⑦. These phages exhibited clear, transparent plaques with distinct edges and no halo on the culture plates (**Figure 1C**). After three rounds of amplification, their titers reached 10^8^ to 10^9^ PFU/mL. Transmission electron microscopy revealed consistent morphological characteristics among these three phage strains, including icosahedral heads (approximately 56 ± 4 nm in diameter) and elongated tails (around 250 ± 18 nm in length) as shown in **Figure 1D**.

### 3.2 Phage CSP1 genome characteristics and evolution analysis

**Figure 2:**
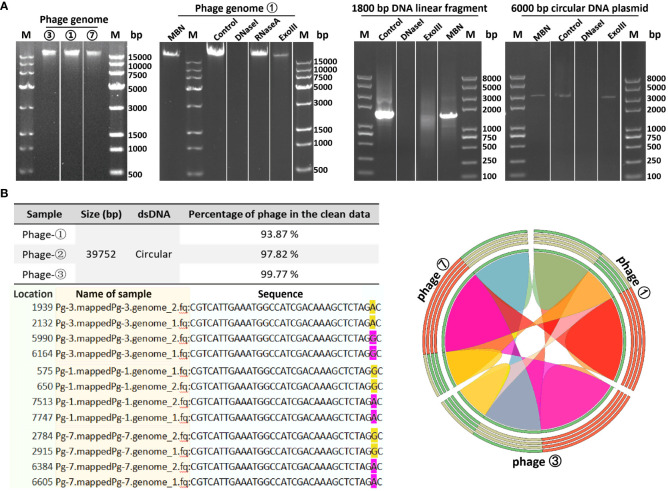
Genomic characteristics of bacteriophages. **(A)** Genomic extraction and enzyme digestion profiles of phage strains. Lane M denotes DL 15000 DNA Marker (500 - 15000 bp)/Trans 2K PlusII marker (100 - 8000 bp). **(B)** Nucleotide comparison (left) and homology analysis (right) of the three phage strains. SNP sites in the sequencing results of phage strains ①/③/⑦ (pg1/3/7) are highlighted in yellow and magenta; the color-coded ribbon diagram displays regions of high homology. Due to the circular nature of the genomes, the start and end positions of the sequences vary.

**Figure 3:**
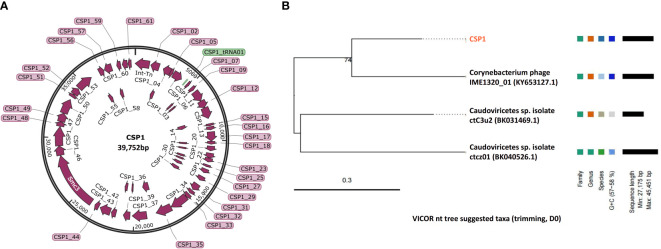
Genomic map and classification of phage CSP1. **(A)** Schematic representation of the circular genome of CSP1. Predicted open reading frames are indicated by magenta arrows, and the tRNA gene is represented by a green bar. **(B)** The nt tree constructed using VICTOR software illustrates the phylogeny and classification of CSP1.

In order to determine the genomic sequences and characteristics of the three bacteriophages isolated from environmental sources, we extracted genomes from three concentrated phage samples (with titers of ① 2.6×10^10^ PFU/mL, ③ 1.8×10^9^ PFU/mL, and ⑦ 2.7×10^11^ PFU/mL) and subjected them to agarose gel electrophoresis analysis (see **Figure 2A**; **Supplementary Figure S1** displays the original image). Qualified samples were then processed for enzyme digestion and genomic sequencing. A series of enzymatic analyses revealed that the phage genomes were digestible by DNase I but not by RNase A, indicating that the extracted genomes were DNA-based; furthermore, the genomes were not completely digested by Mung Bean Nuclease (MBN) and Exonuclease III (Exo III), suggesting a likely double-stranded circular DNA structure. This was contrasted with the control group’s linear DNA, which was fully digested by Exo III, whereas circular double-stranded plasmids remained intact under the action of MBN and Exo III (as shown in **Figure 2A**). Sequencing results further confirmed the isolated bacteriophages as having circular double-stranded DNA genomes (see **Figure 2B**). Homology comparison analysis of these three phage genomes (phage ①/③/⑦) showed that their sequence lengths and structures were 100% identical: with a total length of 39,752 bp, predicting 61 open reading frames (*orfs*) and 1 tRNA (tRNA-Lys) (see **Supplementary Table S1**, **Figure 3** left). Among them, the sequences of phage ①/⑦ were completely identical, with only one nucleotide difference in an unknown functional predicted protein Orf4 in phage ③ (Asp at position 130 in phage ①/⑦, Gly in phage ③) (see **Supplementary Table S1**), suggesting a potential SNP site. Protein encoded by this gene was predicted to be a Transcription regulator (5FD4_B; ComR; Probability 98.06%; E-value 0.000043) using the HHpred server for remote protein homology detection and structure prediction. Consequently, we combined the isolated *Corynebacterium striatum* bacteriophages phage ①/⑦ and named them collectively as CSP1 (*Corynebacterium striatum* phage 1).

To ascertain the taxonomic classification of the isolated bacteriophages, we employed a phylogenetic and taxonomic web service for prokaryotic viruses, conducting a BLASTn search of the CSP1 genome sequence against the NCBI database. The top three species with the highest similarity in NCBI, including phage IME1320_01 (Zhao et al., 2019), *Caudoviricetes* sp. isolate ctC3u2 (BK031469.1), and ctcz01 (BK040526.1), were selected for genome-based phylogenetic and classification analysis using the VICTOR software (Meier-Kolthoff and Göker, 2017). The analysis indicated that bacteriophage CSP1 belongs to the *Caudoviricetes* class, representing a new species distinct from IME1320_01 (as shown in **Figure 3**, right). Additionally, taxonomic prediction based on the phage structural proteins (such as the major capsid protein and portal protein) (Dion et al., 2020) was performed. It was found that the proteins encoded by *orf34* to *orf37* in the CSP1 genome structurally and functionally match the terminase large subunit, portal protein, prohead protease, and major capsid protein of the Enterobacteria phage HK97 (see **Table 1**), further confirming that CSP1 is a new species within the unclassified *Caudoviricetes* class (*Viruses*; *Duplodnaviria*; *Heunggongvirae*; *Uroviricota*; *Caudoviricetes*; *unclassified Caudoviricetes*).

**Table 1:** Predicted HK97-Like structural proteins in the CSP1 bacteriophage genome.

Query (CSP1)		HHpred Hit		
Gene ID (Gene Name)		Hit Enterobacteria phage HK97	Probability (%)	E-value
CSP1_34	*orf34*	6Z6D_A (Terminase Large subunit)	100	6.4e-40
CSP1_35	*orf35*	8FQL_E (Portal protein)	100	6.4e-40
CSP1_36	*orf36*	P49860 PRO_BPHK7 (Prohead protease)	99.93	4e-23
CSP1_37	*orf37*	P49861CAPSD_BPHK7 (Major capsid protein)	100	3e-37

### 3.3 Collinearity comparative analysis of phage CSP1 with other phages

To identify the functional and sequence similarities between the gene clusters on the CSP1 bacteriophage genome and those of other phages or mobile genetic elements (MGE), we conducted a discontiguous megablast search of the CSP1 genome sequence (beginning with the *orf1 integrase* gene and ending with a 20 bp recombination sequence: TGGGCGAATGAGCCGGCCTA) in the NCBI Nucleotide database. The top two sequences with the highest similarity were selected for genomic collinearity analysis: the *Corynebacterium striatum* strain 216 chromosome (Accession no. CP024932.1, spanning from 1172130 to 1210384, 38255 bp) and *Corynebacterium* phage IME1320_01 (Accession no. KY653127.1, 40068 bp). The comparison results indicated the highest sequence similarity between CSP1 and *Corynebacterium striatum* strain 216, with 84% coverage and 95.68% identity, E-value of 0.0. The next closest was the temperate phage IME1320_01, with 46% coverage and 90.71% identity, E-value of 0.0. No other *Corynebacterium* species genomes in the NCBI database were found to harbor CSP1-like prophages.

**Figure 4:**
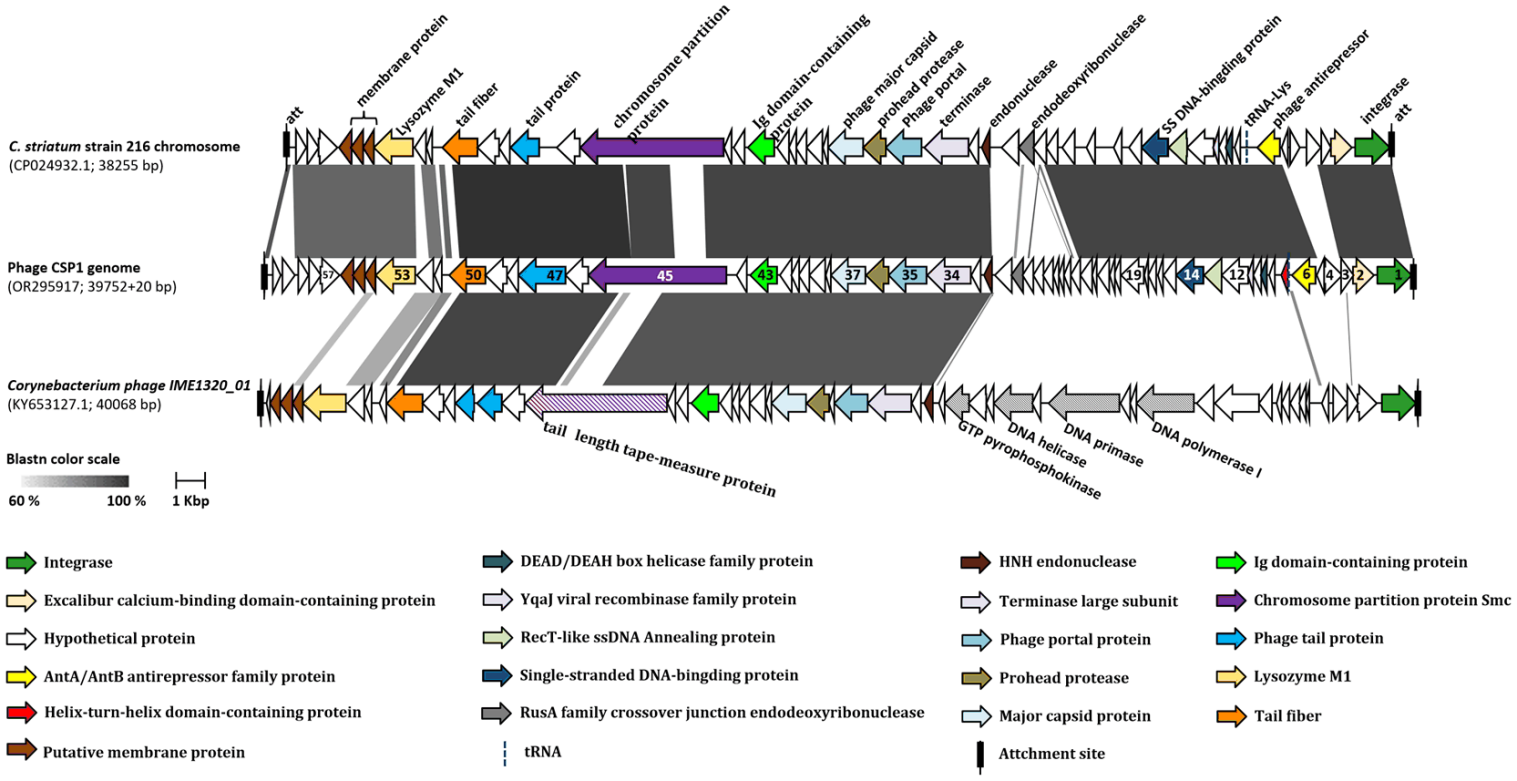
Collinearity analysis of CSP1 with other phages. The ORFs marked with the same colors in the diagram represent genes of similar functions (refer to the legend below the figure). The numbers on the ORFs correspond to the order of their open reading frames. Different shades of grey indicate the nucleotide sequence homology between orthologs.

A collinearity analysis was conducted on the CSP1 genome in comparison with the MGE (prophage) on the *Corynebacterium striatum* 216 chromosome and the *Corynebacterium* phage IME1320_01 (**Figure 4**). It was found that CSP1 shares a high degree of similarity with the *C. striatum* 216 MGE in terms of integrase and terminal recombination attachment sites (*att*). Furthermore, the arrangement of gene clusters was also strikingly similar, including lysogeny-related genes, phage antirepressors, and structural and packaging gene modules (refer to **Figure 4**; for protein sequence comparison, see **Supplementary Table S2**). The genomic organization of CSP1 also showed similarities with the *Corynebacterium* temperate phage IME1320_01 in aspects of phage integration, structure, and packaging, although there was a lower degree of similarity in the nucleic acid replication module (see **Figure 4**; **Supplementary Table S2**). Overall, CSP1, along with *C. striatum* 216 prophage and the IME1320_01 genome, exhibited characteristic genes of the *Siphviridae* family, indicating that their tails are composed of a central tape measure protein surrounded by a tail tube, ending with a terminator protein (Arnaud et al., 2017).

### 3.4 Host range of phage CSP1

**Figure 5:**
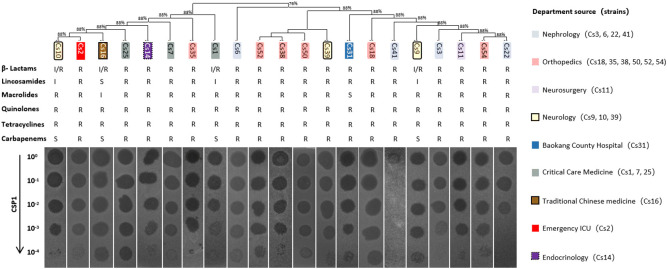
Host range of CSP1. The figure presents the phylogenetic and antibiotic resistance profiles (R, resistant; S, susceptible; I, intermediate) of clinical multi-drug resistant *Corynebacterium striatum* (MDR-*Cs*1 to *Cs*54) hosts, labeled above the phage dilution spot assay diagram. The corresponding departments of origin for the host bacteria are indicated in the legend on the right.

To determine the host range of the bacteriophage CSP1 isolated from environmental sources, 54 strains of MDR-*C. striatum* (Wang et al., 2022) and other bacteria including *Escherichia coli* (DH5α, DHM1, BL21), *Staphylococcus aureus*, *Acinetobacter baumannii*, and *Enterococcus faecalis* were tested as potential hosts. The dilution spot assay with phage CSP1 on culture plates of these strains resulted in plaque formation on 21 strains of MDR-*C. striatum*, while no plaques were observed on the remaining 33 strains of MDR-*C. striatum, one strain of C. pseudodiphtheriticum*, and the 6 strains of non-*Corynebacterium* control bacteria (see **Figure 5**; **Supplementary Figure S2**). Notably, CSP1 infected the *Cs*41 strain from the Nephrology department, but plaques disappeared after dilution. Further analysis of the susceptible clinical isolates revealed their distribution across nine departments, with Nephrology (4 strains), Orthopedics (6 strains), Neurology (3 strains), Critical Care Medicine (3 strains), and one strain each in Neurosurgery, Traditional Chinese Medicine, Emergency ICU, and Endocrinology (Wang et al., 2022). There was no clear correlation between the phylogenetic relationships of these sensitive strains and plaque formation (see **Figure 5**). The 21 CSP1-sensitive strains exhibited a 100% resistance rate to β-lactams, quinolones, and tetracyclines, with over 80% resistance to other classes of antibiotics (see **Figure 5**).

### 3.5 Physiological characteristics of phages

**Figure 6:**
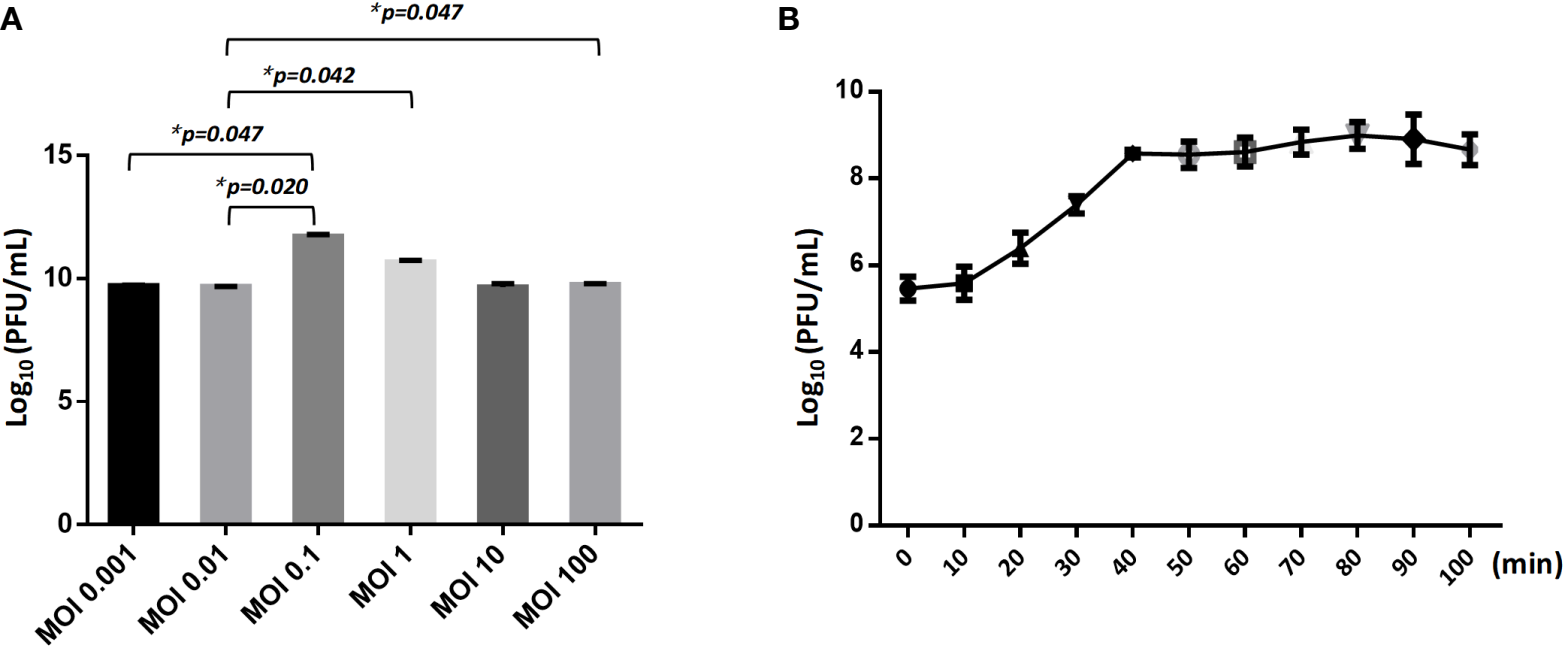
The MOI and One-Step Growth Curve of CSP1. **(A)** The influence of different ratio of bacteriophages and bacteria in suspension (MOI) during CSP1 multiplication (*p < 0.05, was considered statistically significant). **(B)** One-step growth curve of CSP1 propagated in *Cs*11. Means and standard errors from three independent experiments are shown.

**Figure 7:**
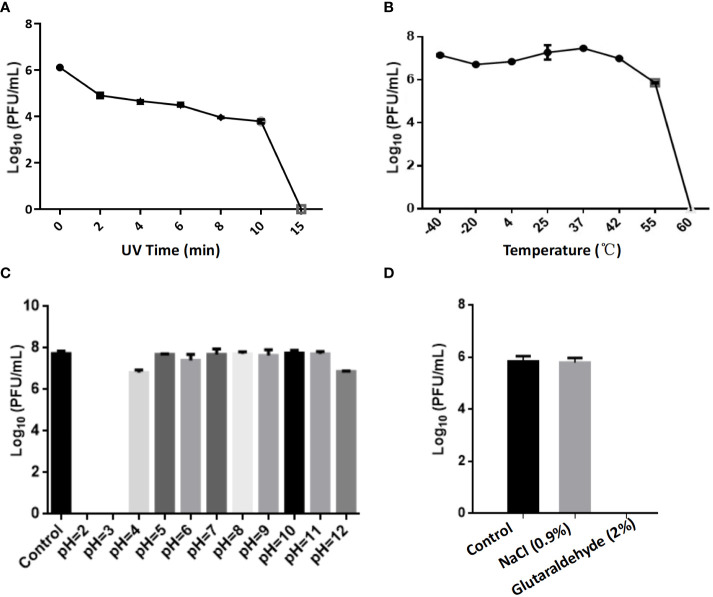
The Impact of Various Physicochemical Factors on the Stability of Phage Potency. **(A)** The effect of ultraviolet (UV) irradiation for varying durations on phage potency. **(B)** Analysis of phage potency after incubation at different temperatures for 1 hour. **(C)** Analysis of phage potency after incubation in culture media with varying pH levels for 1 hour. **(D)** Analysis of phage potency after incubation in solutions of sodium chloride (physiological concentration of 0.9%) and glutaraldehyde (disinfectant concentration of 2%) for 1 hour.

The optimal Multiplicity of Infection (MOI) for phage CSP1 was determined to be 0.1, as depicted in **Figure 6A**. Following incubation at 37°C for 8 hours, the highest titers recorded were 6.37×10^11^ PFU/mL, 4.84×10^11^ PFU/mL, and 4.77×10^11^ PFU/mL, respectively (refer to **Supplementary Table S3**). Analysis of the one-step growth curve revealed that CSP1 has a latent period of less than 10 minutes, followed by a lysis phase lasting approximately 30 minutes before reaching a plateau phase, as shown in **Figure 6B**. During this phase, the release of phage CSP1 reached a titer of 10^9^ PFU/mL (**Supplementary Table S4**). Stability tests for CSP1 demonstrated a complete loss of activity after 15 minutes of UV irradiation, with a reduction of 2-3 log scales within the first 10 minutes (**Figure 7A**). Temperature sensitivity assays indicated that CSP1 maintained its titer stability when incubated at temperatures ranging from -40°C to 52°C for 1 hour. However, there was a one-log reduction at 55°C, and complete inactivation occurred at 60°C (**Figure 7B**). In pH stability tests, CSP1 was found to be inactive at pH 3 and below, stable between pH 5-11, and exhibited a one-log reduction in titer at pH 4 and pH 12 (**Figure 7C**). When incubated in 0.9% NaCl and 2% glutaraldehyde solutions for 1 hour, CSP1 maintained its titer stability only in the sodium chloride solution, while losing its activity in the 2% glutaraldehyde solution (**Figure 7D**).

**Figure 8:**
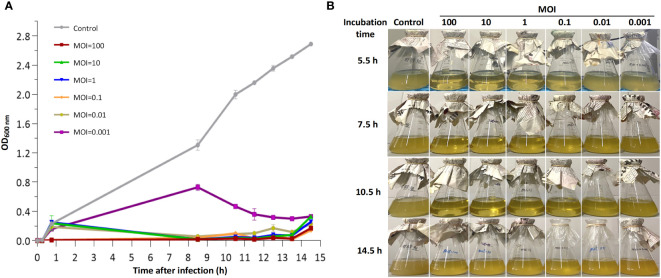
*In vitro* bactericidal activity of phage CSP1 against *C striatum* strain 11 (*Cs*11). *Cs*11 was infected with CSP1 at MOIs of 100, 10, 1, 0.1, 0.01, and 0.001 and cultured for up to 14.5 hours. **(A)** Lytic curves of the phage. **(B)** Presentation of phage and bacterial cultures. The control group represents bacterial cultures without the addition of phage. Data are presented as means ± SD (error bars) from three independent experiments.

To evaluate the *in vitro* bactericidal activity of phage CSP1, the MDR-C. striatum strain 11 (Cs11) was cultured to early logarithmic phase (OD_600 nm_ = 0.22) and then inoculated at 1% into fresh LB liquid culture. It was subsequently infected with CSP1 at various multiplicities of infection (MOI) at 37°C with aerobic orbital shaking. As illustrated in **Figure 8**, the vast majority of Cs11 cells were eradicated by CSP1 within 10 hours of infection at MOIs greater than 0.001. Beyond 12 hours, bacteriophage-resistant bacteria began to emerge across all MOI conditions in the mixed cultures (**Figure 8A**). By 14.5 hours, a significant growth of phage-resistant bacteria was evidently observed in the culture conditions (**Figure 8B**).

### 3.6 Cell safety of CSP1

**Figure 9:**
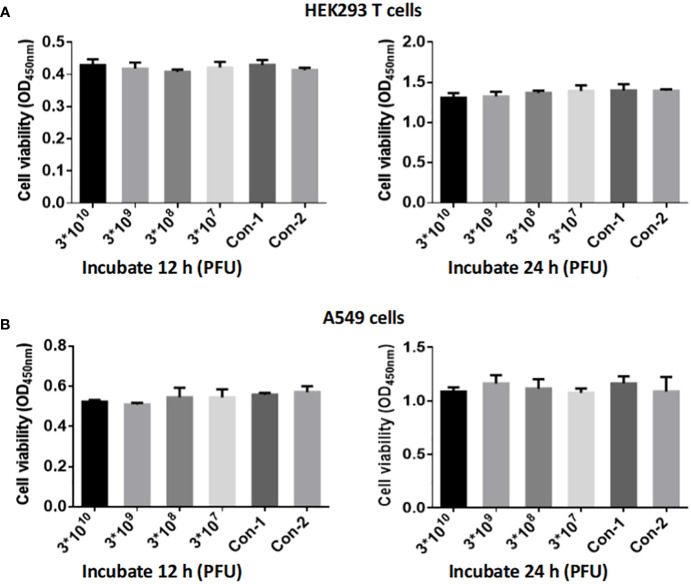
Evaluation of CSP1 Phage Cytotoxicity on HEK293 T **(A)** and A549 Cells **(B)**. Cells were exposed to varying concentrations of phage CSP1 (3×10^12^, 3×10^11^, 3×10^10^, 3×10^9^ PFU/mL) for durations of 12 h and 24 h. Control conditions included Con-1 with 10 µL of LB medium and Con-2 with 10 µL of standard cell culture medium. Results are presented as mean ± SD from three independent experiments.

Different titers of phage CSP1 were incubated with HEK293 T and A549 cells for 12 hours and 24 hours, respectively, to evaluate the cytotoxic effects of CSP1 using the CCK-8 assay kit for cell viability measurement. As illustrated in **Figure 9**, both HEK293 T and A549 cells incubated with CSP1 showed consistent cell viability compared to control groups 1 (10 µL LB medium) and 2 (10 µL cell culture medium), regardless of the incubation duration (12 or 24 hours), with no statistically significant differences observed (**Supplementary Table S5**). Additionally, the titer of phage in the co-culture medium of cells was determined (**Supplementary Table S6**), revealing no statistically significant difference in phage titer before and after incubation, suggesting that CSP1 does not enter the cells, which aligns with the specificity of phage recognition. These results indicate that the isolated and purified CSP1 phage is non-toxic, laying a foundation for future *in vivo* antimicrobial therapy research.

## 4 Discussion

We are currently in an era where antibiotic resistance is declared by the World Health Organization as one of the greatest threats to global health, food security, and development (Shrivastava et al., 2018). Among all potential alternatives to address this urgent need, phage therapy appears to be one of the best options (Hatfull et al., 2022). The study of natural bacteriophages targeting the emerging opportunistic pathogen *Corynebacterium striatum* has been limited. This study reports for the first time a novel phage isolated from hospital sewage, named CSP1, capable of lysing clinical multi-drug resistant *Corynebacterium striatum*. CSP1 possesses an icosahedral head (56 ± 4 nm) and a long non-contractile tail (250 ± 18 nm) (**Figure 1**), typical of the *Caudoviricetes* class (Zhu et al., 2022), with its genetic material being circular double-stranded DNA, the most common type used in therapeutic phages (Petrovic Fabijan et al., 2020). The genome structural genes and assembly protein function predictions (**Table 1**) indicate that CSP1 has a capsid protein folding structure and portal protein and capsid assembly pathway similar to HK97 phages (Rixon and Schmid, 2014).

Fortunately, although CSP1 shares genomic organization similar to the inducible temperate phage IME1320_01 (Zhao et al., 2019), such as lysogeny-related genes and phage structural and packaging gene clusters (**Figure 4**; **Supplementary Table S2**), it demonstrates a broader host spectrum by infecting and lysing 21 different clinical MDR-*C. striatum* isolates (**Figure 5**). The one-step growth curves, optimal MOI, and stability results showed that the phages displayed relatively short latency periods (less than 10 minutes) and a lower MOI (0.1), with broad pH (4 to12) and thermal stabilities (-44 to 55°C) (**Figure 7**). This implies that fewer CSP1 is required to rapidly absorb to the host surface and lyse more bacterial cells within a certain period, demonstrating the potential as a stable biological agent suitable for biocontrol applications (Fang et al., 2023). Although phages with a broader host spectrum are generally more desirable, CSP1 did not affect more than half (33 out of 54) of the clinical MDR-*C. striatum* isolates tested. Among the 21 sensitive strains, CSP1’s infection of the *Cs*41 strain was peculiar, showing only weak plaques at high phage concentrations in the dilution spot assay (**Figure 5**). This may relate to bacterial variation, as differences in phage infection characteristics exist among clinical isolates within the same species (Hatfull et al., 2022). The mechanisms behind these differences could be further investigated by high-throughput whole-genome sequencing of all 54 *Corynebacterium striatum* strains, with a focus on phage infection-related genes (Pope et al., 2011) and potential anti-phage defense mechanisms (Marraffini, 2015), thereby potentially expanding the host range of CSP1.

It is well-known that phages are categorized into lytic (virulent) and temperate types (Hatfull et al., 2022). Strictly lytic phages have been widely recognized in phage therapy (Monteiro et al., 2019). In contrast, temperate phages are avoided due to their inherent capability to mediate gene transfer between bacteria, potentially increasing bacterial virulence, such as promoting antibiotic resistance (Farrugia et al., 2015). Genome sequencing and collinearity analysis of CSP1 showed high DNA sequence and gene cluster arrangement similarity with the temperate phage IME1320_01 and *C. striatum* strain 216 prophage. They all possess lysogeny-related gene elements, especially CSP1 and *C. striatum* strain 216 prophages showing high similarity in *integrase* (Identity 93.5% & similarity 97.5%) and identical recombination core sequences (**Figure 4**), suggesting that CSP1 might be the first temperate phage isolated from the environment targeting *Corynebacterium striatum*, capable of site-specific integration under the action of phage-encoded Integrase (Orf1), recognizing *attP* on the phage and *attB* on the bacterial chromosome (Wang et al., 2018). Interestingly, in the host range screening experiment of this study, turbid plaques typical of temperate phages were observed on the *Cs*41 agar plate (**Figure 5**). Moreover, as incubation time increased, a thin bacterial lawn formed within all phage plaques (**Supplementary Figure S2**), suggesting the emergence of lysogenized or phage-resistant bacteria. This observation is consistent with lytic assay findings (**Figure 8**), where CSP1’s lytic activity halted after 12 hours of incubation, leading to substantial growth of phage-resistant bacteria (Chen et al., 2020). Future work will utilize bioinformatics and PCR amplification to identify specific recombination elements in clinical MDR-*C. striatum* isolates. Pinpointing the genes governing CSP1’s lysogeny and lytic capabilities is crucial for the engineered modification of phages, paving the way for their application in bacteriophage therapy (Yosef et al., 2015).

Phages, as viruses of bacteria, possess a series of unique genes. In this study, the CSP1 genome is predicted to encode 61 *orfs* and one *tRNA*. Most *orfs* encode proteins of unknown function, and no virulence or pathogenicity-related genes have been found; *orf4* (CSP1_04; predicted as a transcription factor by HHpred) has an SNP site, and further studies using genetic engineering techniques (such as CRISPR-Cas9) to knock out or insert target genes, constructing phage mutant strains, will explore the differences between mutant and wild-type phages in host bacterial infection and observe the impact of transcription factors on the phage life cycle (Guan et al., 2022). Additionally, a “treasure” gene (*orf53*) is noted, predicted to encode lysozyme M1 (1,4-beta-N-acetylmuramidase, CSP1_53; **Figure 4**; **Supplementary Table S1**), one of the five types of endolysins in phages (Gondil et al., 2020; Ramesh et al., 2022), capable of hydrolyzing the 1,4-beta linkages between N-acetyl-D-glucosamine (GlcNAc) and N-acetylmuramic acid (MurNAc) in the peptidoglycan heteropolymers of bacterial cells (Davies and Henrissat, 1995). Future studies could focus on gene cloning, protein expression and purification, enzymatic analysis, and structure-function research to develop novel antibacterial treatments (Abdelrahman et al., 2021).

## 5 Conclusions

This study is the first to report CSP1, a naturally occurring phage targeting the clinical MDR-*C. striatum* isolates, isolated from the environment. Through the isolation, identification, genomic characterization, evolutionary analysis, host range screening, physiological properties, and cell safety research of this phage, we discovered that it possesses an icosahedral head and a long, non-contractile tail, categorizing it as a new species within the unclassified *Caudoviricetes*. Its circular double-stranded DNA genome, spanning 39,752 base pairs, is predicted to encode 61 open reading frames (*orfs*) and 1 tRNA, with no genes associated with virulence or pathogenicity identified. Among these, *orf4* (CSP1_04) is an uncharacterized gene with an SNP site; *orf1* (CSP1_01) encodes an integrase belonging to the tyrosine family; and *orf53* (CSP1_53) encodes a lysozyme. Additionally, beyond lytic and lysogenic genes, the CSP1 genome contains a complete set of structural and packaging gene clusters, characteristic of HK97-like phages. CSP1 demonstrates a broad host range, capable of infecting and lysing 21 different clinical multidrug-resistant *C. striatum* isolates from various departments (20 forming clear plaques and 1 forming turbid plaques), indicating its potential as a temperate phage with both lytic and lysogenic life cycles. Furthermore, as a Gram-positive bacterium phage, CSP1 exhibits significant environmental adaptability and biosafety: maintaining stable efficacy from -40 to 55°C, pH 4 to 12, and in 0.9% NaCl buffer solution, without exhibiting any cytotoxicity. In summary, this study lays a crucial foundation for future exploration and modification of *C. striatum* phages and their derivative endolysins as novel antimicrobial resources.

## Data availability statement

The datasets presented in this study can be found in online repositories. The names of the repository/repositories and accession number(s) can be found in the article/**Supplementary Material**.

## Ethics statement

This study follows internationally recognized ethical principles, such as the Declaration of Helsinki, the international ethical guidelines for biomedical research and other relevant policies. It was approved by the Scientific Ethics Committee of Hubei University of Arts and Science (Decision no. 2022-023).

## Author contributions

JW: Conceptualization, Funding acquisition, Investigation, Methodology, Project administration, Software, Validation, Writing – original draft, Writing – review & editing. MZ: Investigation, Validation, Writing – original draft. JP: Investigation, Validation, Resources, Writing - original draft. WY: Investigation, Validation, Writing – original draft. LF: Funding acquisition, Investigation, Software, Validation, Writing – original draft. CW: Methodology, Resources, Writing – review & editing. XX: Resources, Supervision, Writing – review & editing.
