# Supplementary material for: Isolation and identification of a novel phage targeting clinical multidrug-resistant Corynebacterium striatum isolates
Source: Front Cell Infect Microbiol. 2024 Mar 20;14:1361045. doi: 10.3389/fcimb.2024.1361045 (PMC10987712; doi:10.3389/fcimb.2024.1361045)
Supplement: Supplementary file 1 [file DataSheet_1.pdf]

Supplementary Material

1 Supplementary Figures and Tables

1.1 Supplementary Figures

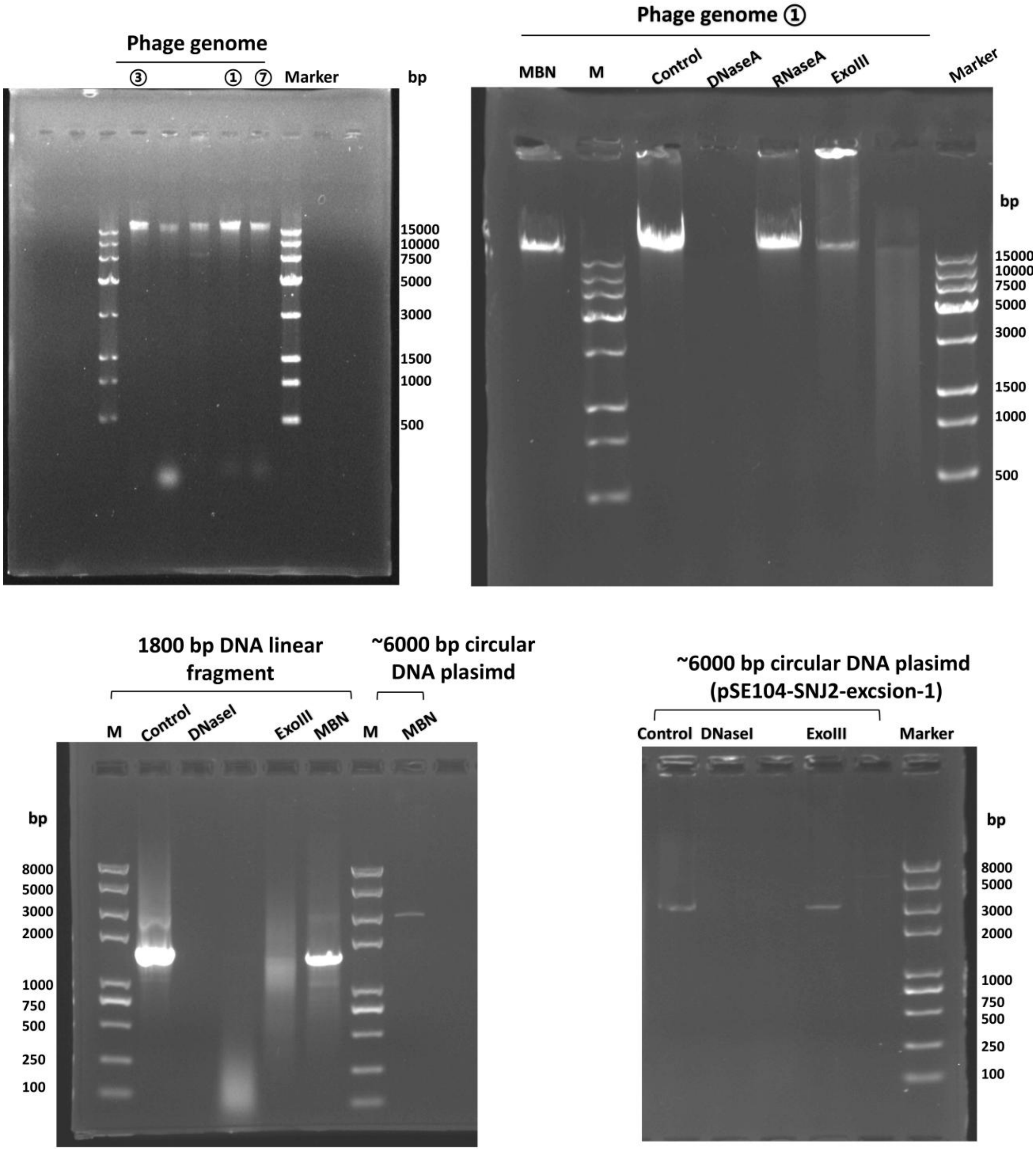

Supplementary Figure 1. Electrophoresis gel image of phage genome extraction and enzymatic digestion identification.

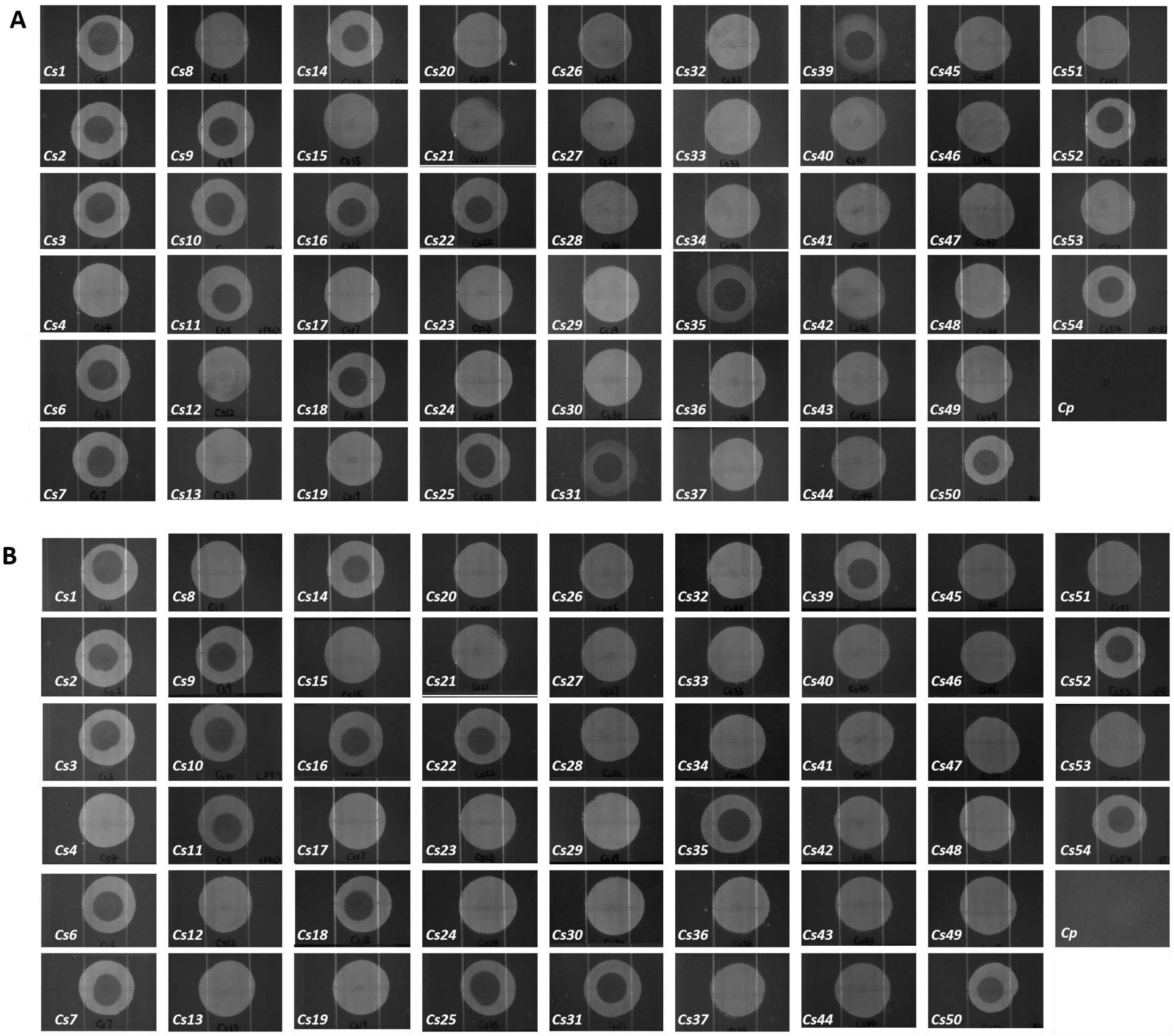

Supplementary Figure 2. The figure presents the effects of inoculating bacteriophage CSP1 (5 µL, titer approximately 3.4×10^{8} pfu/mL) onto the soft agar cultures of 52 *Corynebacterium striatum* isolates, with an approximate diameter of 1.9 cm (strains *Cs*-5 and *Cs*-38 were not included). (A) Post-incubation at 37°C for 12 hours (*Cs*-1, 2, 3, 4, 6, 7 incubation for 17 h). (B) Post-incubation at 37°C for 18 hours. *Cp*, *Corynebacterium pseudodiphtheriticum* clinical isolate incubation for 12 h (A) and 36 h (B).

1.2 Supplementary Tables

Table S1. Genomic Annotation Information of Bacteriophage CSP1

Gene ID	Strand	Location (Start - End)		Gene Name	Annotation	CDS_seq	Protein
CSP1_01	-	1191	1	*orf1* Int-Tn	tyrosine-type recombinase/integrase	ATGGCAGTCCAGAAACGCAGAACTAAAGCCGGTAAAACAGTTTATGTCGCACGATGGCGCGACCCGTCCGGAAAAGAGCACTCAAAGAGCTTCCCGCTTGCCCGCGAAGCTAAAGCGCACGTCCAGGAGATGGAGCGCGCCACACGAATCGGAATAAATCCTACGGCTGGGGACAAGGTGACCGTCCATGAACTCATGACCTTATGGATTGAGGACCGCAACATACGCCCGGCAAGCCTCGCTCTTTACACGCGTACCCGTGATAAGCACCTGGGGCCGCTGGCAGAGTGGCCCGCGAAAGACCTCACCCCGCAGGCCGTCCGCGACTGGCAAACCCAACTCCGCACATGCCGGTCGTGGCTGTCCAAAGATGATACGGGCATGTCGCCTAGGTATGTGCAGACCGCCGCTAGCCACCTCCGCTCCGCCTATAAATGGGGCATCGAGAATGAGTTGGTGGCGAAGAACCCGGTTCGGGTCGAGCGTGTACAGGCCGCGGTGGAGCCGGACGATGTGCCGACAATGAAAGAAATTCAGGCGGTTATCGACCGGGCGCGTGCCGGCGGTGCGGCGTACACAGAGGTTAAGACCAAGGGTGAGAAGCCGCGCAAGCTGGTGTTGCGCCCGGACCCCATGTTTGCGGACATGCTCACGGTAGCGGCTTTCACCGGCCTGCGTATTGGTGAGCTGTGTGGGCTGCTGGTCGAGGAGGTCAGCCTCGACTTGGGTGTGGTGCAGGTGCGTAAGCAACTGGATAAGCGGCCTCCGCATGGGCGTGTGGCGTTGAAGTCGAAGAATTCACGCCGGGATGTGCCTATCTCTTCCGAGCTGCGCCCGGTGCTGGAAAGGCTGACCGCTGGCCGTATGCCGGGCGAGTACCTTTTCACGAATCGGAATGGCCGGCCATTCCTGCCATCCGGAGCCGGGGATAAGGTGCTGCGGGTTGCAACATTCGAGAAAGCACCCAGGGTGCATTTCCACGCGTTGCGTCACCACTTCGCGAGCTCCCTACTTTTGGCCGGCGTTCCGGTGCCGGACGTAGCGCGAGTGCTGGGGCATACCGCGGCGCAGCTGTTGAAGACGTATGCGCATGCGCTGCCGGGTAGTGGGGATCGTGTGGCGGCTGCTATCAGTGCGTCTGTGGGGTGCGGGATTTCTGCGGGATCGCCGCACCTGAGGGTGGTTGGCTAG	MAVQKRRTKAGKTVYVARWRDPSGKEHSKSFPLAREAKAHVQEMERATRIGINPTAGDKVTVHELMTLWIEDRNIRPASLALYTRTRDKHLGPLAEWPAKDLTPQAVRDWQTQLRTCRSWLSKDDTGMSPRYVQTAASHLRSAYKWGIENELVAKNPVRVERVQAAVEPDDVPTMKEIQAVIDRARAGGAAYTEVKTKGEKPRKLVLRPDPMFADMLTVAAFTGLRIGELCGLLVEEVSLDLGVVQVRKQLDKRPPHGRVALKSKNSRRDVPISSELRPVLERLTAGRMPGEYLFTNRNGRPFLPSGAGDKVLRVATFEKAPRVHFHALRHHFASSLLLAGVPVPDVARVLGHTAAQLLKTYAHALPGSGDRVAAAISASVGCGISAGSPHLRVVG
CSP1_02	-	2026	1307	*orf2*	excalibur calcium-binding domain-containing protein	ATGACTTCTAATCCCTTTTCTCCTGGACCCCAGCATAAGGAGCCCGCTCGATGGAAGGGCATCTTGGCGTGGGTAGCGGTTATCTTCGGTGTTTTAATGATTATTGCGAGCCTTGCCAGTGGTACTCTCCGTGACACATTGGGCGGATTTTTCAGCGGCGTAGCCCTCATGCTCCCCGGCGCCTGGTACATCCTCCACAACCGCCGAGAGAAGAACGGCGCTGTCCCAATGAAGCGTCACTGGGGACTAATCACAGTAGCTTCAATCACGCTGCTCATTCTTGCCGGCGTAACAGCGCCAGCCAAGAGCCCAGATGAAGGCGACAGCGCCGTTGAGGAATCCTCGACAACCGCAGCGCCCAGTTCTACGACGTCGCCTGAGCAAACGTCTTCTTCTAAGGTGACCGCTTCATCGGAACCGACAACGCCGCCGCCAGCTCCTTCACCTACCCTCGAAGAACAGGGTGAGAGCGAGGAGAACAACGATGTTGACGCATACCAAGTCGCGCCGCCAGCCCCGGCGCCGGAAGTCTCCTACGAGGAGCATCCCGTCTACGAGGAAGCGCCGGCTCCTGCACCTGCACCCGCGCCTCAGTACTCGGGAGGTAGTGGCCGAACCTGTGCAGAAATTGGCCACAAGGTCTACCCGGGCGATCCGGAATATTCACTCGAGCGAGACAAGAACGGTGACGGCGTTGGCTGCGAATCCCAACCAGGCTAA	MTSNPFSPGPQHKEPARWKGILAWVAVIFGVLMIIASLASGTLRDTLGGFFSGVALMLPGAWYILHNRREKNGAVPMKRHWGLITVASITLLILAGVTAPAKSPDEGDSAVEESSTTAAPSSTTSPEQTSSSKVTASSEPTTPPPAPSPTLEEQGESEENNDVDAYQVAPPAPAPEVSYEEHPVYEEAPAPAPAPAPQYSGGSGRTCAEIGHKVYPGDPEYSLERDKNGDGVGCESQPG
CSP1_03	-	2451	2023	*orf3*	hypothetical protein	ATGAAGATGCTTTGCGTGAAGAAGGTGATTGGACTGACCCGGATTACATCCCCTGACCTCCACCGGCTCGCCGAATCGATGGGGGTCACCCTCACCCGGCACACAGGCGGTGAGAAAGGCTGGTATGACCACGCCACACGCACGATTAGCACCCGGCGCGGCATGAGCATCGCCCAATACCGCTCCACCCTCGCCCACGAGCTAGGCCACGCACACTACGGTGACACCCCCACCGGGAACGGCCACTACGACCAACGCCAAGAAAACCGAGCCTGGGCGTACGCCGCAAACCTACTCATCTCCCCCGTAGACTTCGCAGCCGCCGCACTCTGGCACAACGACAACGTGCCGGCCATCGCAGACGAACTCGAAGTCACACAGCACCTACTCCGAACTTGGATCGCTCTCTACGAAAGGACAGCGGCATGA	MKMLCVKKVIGLTRITSPDLHRLAESMGVTLTRHTGGEKGWYDHATRTISTRRGMSIAQYRSTLAHELGHAHYGDTPTGNGHYDQRQENRAWAYAANLLISPVDFAAAALWHNDNVPAIADELEVTQHLLRTWIALYERTAA
CSP1_04	-	3037	2396	*orf4*	hypothetical protein	GTGAGCCTCCTAGTTTCAATCTTTCGCCGGCTTGAACCGGGCAAGATTCATTATGAACCTATCAAGGTACATTGTCAACCTATTTCGGTTCATTCTTCTAATTTCGCAGCTCTTGACACCTATCCCGTAATGGTTCATAATGAACCTATGAACATTGAAGAATGGCTACATACACTCCCCGGCTCCCCTACCGTGAGCCAAGCGGCAGACCATACCCCTGGCGTATCTAAAGCAACGTTTCTCCGTCATGCCGACAAAGGGAAAACAACCGCCGAATACGTCATCGCTATCGCCCGCGCCCACAACGTCAATGAAATTCAAGCGCTCGCAGACCTGGGTTTCGTCGAAGAAGCAGCCGTCATTGAAATGGCCATCGACAAAGCTCTAGACATGGCCACCAACTCCAAACTCCTAGAGGAAATCAACAAACGAGTAGACCCCGAAGCCCGACGCCTTTTCCACGGCGATGGAGTACCAGGCATCATCGACGTTCGCGATGACGAACTGGCCACACGCCGTCAAAACACCCCCGTGTCCGGCCCGTCTGATTCAATGCCCCTGTCGGCGGTTGCTGATGATTCCCCTGATGAAGATGCTTTGCGTGAAGAAGGTGATTGGACTGACCCGGATTACATCCCCTGA	MSLLVSIFRRLEPGKIHYEPIKVHCQPISVHSSNFAALDTYPVMVHNEPMNIEEWLHTLPGSPTVSQAADHTPGVSKATFLRHADKGKTTAEYVIAIARAHNVNEIQALADLGFVEEAAVIEMAIDKALDMATNSKLLEEINKRVDPEARRLFHGDGVPGIIDVRDDELATRRQNTPVSGPSDSMPLSAVADDSPDEDALREEGDWTDPDYIP
CSP1_05	+	3038	3283	*orf5*	hypothetical protein	ATGATCGATACCCGTCCAGTATCTGCTTATAGCTTCCGCCCTGGTGCGCTAGAGCACATCATGCGCTCCCGCAACCTGCGAACCGACAAACAGCTCGCAGCAGCTTTGGGAGTCAGGCTAGATGACCTGGCAGAGATTCGGGCTGGAGCTCCAGTATCTGCCCGACTCGCGCTCAAGGTCAGCGCACTACAGGGCGATGAAAAGTACATAGCAGCATGGTTCGACCCCTACGAACCCGCCGCATAG	MIDTRPVSAYSFRPGALEHIMRSRNLRTDKQLAAALGVRLDDLAEIRAGAPVSARLALKVSALQGDEKYIAAWFDPYEPAA
CSP1_06	+	3334	4140	*orf6*	antA/AntB antirepressor family protein	ATGGGTGAACTAATTCCCATTCAAGACCATGCCGGCATTCAGGCAGTCATGGGCCGAGACCTCTATGACTTCCTCGGTGTTGAGACGGAGTACCGGCACTGGTTCCCGCGCATGGTCGAATACGGCTTTGAAGAGGGCAAAGACTACGTGGTCAAAAATGACCGGGTACAAGATTCCCTCGGACGCAGCCGGGAGCGAACCAACCACATCGTGTCTCTGGACATGGCTAAGGAAATTTCCATGATTCAGCGTACGGACAAAGGTAAGCAGGCGCGCCAGTATTTCATCGAATGCGAGCGCCGGGCGAAGGATCCAGCGCAGCTGTCCCCGGAAGAGCTCATGGCCCGCGCCATCAAGGTTGCTGACAGCACCATCAAGGAGCTCGAAGCAAAGACCGCAGACCAGGCCCTTGCACTCGAAGCCGCGAAGCCAAAAGTGGAGTACTACGACCACTGCGTATCCATCGAGTCCGACGTCATGACCGTTAAAGACTGGGGCGCACACTTCGGGCTGACTGAGCCACAAGCGCGCCAGAAACTACTGGACGCAAAAATGATTTACCGCAAGGTTCAGACCCGTGAGTGGTCATCCCGCAAGGGTGACGTGGTTGACCGCAACGAGTACCGCGCCTATGCCGCCTACCGCAATCTGTTTGACCTGCGTGCCCAACACAACGCTCCGCGCTACCACAACGGGCAGGTGCGTCAAACGCTGTACGTGCGTGCTGGGTGGGCAATCACCGTAGCCAAGAATGCCGGCATTGAGGTGGACCGCTCTATCTATGACATCTTGCCGGAGTCTGTCTGA	MGELIPIQDHAGIQAVMGRDLYDFLGVETEYRHWFPRMVEYGFEEGKDYVVKNDRVQDSLGRSRERTNHIVSLDMAKEISMIQRTDKGKQARQYFIECERRAKDPAQLSPEELMARAIKVADSTIKELEAKTADQALALEAAKPKVEYYDHCVSIESDVMTVKDWGAHFGLTEPQARQKLLDAKMIYRKVQTREWSSRKGDVVDRNEYRAYAAYRNLFDLRAQHNAPRYHNGQVRQTLYVRAGWAITVAKNAGIEVDRSIYDILPESV
CSP1_07	+	4744	4938	*orf7*	helix-turn-helix domain-containing	ATGAATCCTAGCAAAAAGGCCCGAACGTACTCTGTGGCCGAAACATCCGAAATCCTTGGGGTAAGTACCCGCTCGCTTTACCGCCATGTGAAAAGCGGTGCGGCTGCGCACTTGCACCCGATCACTGTTGGTGACCGCGTTGTCTTCCCGCGCCGCGTCATCGATTCCCTAGTGGAGCCGGCGGTGTCCGC	MNPSKKARTYSVAETSEILGVSTRSLYRHVKSGAAAHLHPITVGDRVVFPRRVIDSLVEPAVSA

					protein	ATGA	
CSP1_08	+	5018	5239	*orf8*	hypothetical protein	ATGACCTCCCAGCTTTCAACCACGACGCGTGAGGATCTTCTCGCCGTT<br>GCGCGCGGTAAGCGGATCCGCAATAAAACCCGCCTGCATGAACTCGA<br>ACGCCTGGGCCTGGTTGCCCTGACGACTGTGAGCCATGAACTTCGCAT<br>TCGCGAGATCACCAGCGAGGGCATGGCAGCGCTTACTGCGATGGGGA<br>AGGAGGGGCGATGGATGCCGCGCAAATTCTAG	MTSQLSTTTREDLLAV<br>ARGKRIRNKTRLHELER<br>LGLVALTTVSHELRIREI<br>TSEGMAALTAMGKEGR<br>WMPRKF
CSP1_09	+	5218	5481	*orf9*	DEAD/DEAH box helicase family protein	ATGGATGCCGCGCAAATTCTAGACACGCTGGTGTGGGCGGCGCTTAC<br>CCTTGTCTGCCTGCTGAACTTTCTGCTCATCCGCCGCGTATCACGCGA<br>CCTTGATGCCCTGTGGGACTACCTCGGGCGACAACACCGCCTCATGGC<br>GAACTTGATAGCCCCAAGCGAAGAACCGAGAGCTGAAAAGACGCCG<br>AAGCCTACCGTGCAGGCGGGCCACTTTGACGAGCTTTTAGAAAAGCT<br>CAACCAGCCGAAAGGCCGCCGCGCATGA	MDAAQILDTLVWAALT<br>LVCLLNFLLIRRVSRDL<br>DALWDYLGRQHRLMA<br>NLIAPSEEPRAEKTPKPT<br>VQAGHFDELLEKLNQP<br>KGRRA
CSP1_10	+	5478	5678	*orf10*	hypothetical protein	ATGACATGCATGACGCCTATTCGTCTTGAAAGCAAATCCATTTTGGTT<br>GAGGAATTCAATTTTTTGCGCAGCTGTGGAGACCCTGTCGCTGCCTCC<br>ATCTTCCACCTCGCCAAGGTCTACGGCCTGACTTATTCGACCGTCGAG<br>ACCTACCTCAGCCGCAATCGAAAGAAACTTCACTATGACATCACGTAT<br>TCTCGCTAA	MTCMTPIRLESKSILVE<br>EFNFLRSCGDPVAASIF<br>HLAKVYGLTYSTVETY<br>LSRNRKKLHYDITYSR
CSP1_11	+	5656	6576	*orf11*	YqaJ viral recombinase family protein	ATGACATCACGTATTCTCGCTAACCCACCCGCGCCTGGAACCGCGGA<br>GTGGGCGAAAATTGTTACGGCGTCGAAAGCTCCGGCGTTGCTCGGCG<br>TCTCGCGTTTCACCTCGCAGTATGCCGCGTGGCACCAGCTCGCCGGGT<br>TGATTGACGTTGACATGATGGACCCGGATCGCGCCGCGTGGGGTCAT<br>ATTGCCGAGGCCTCCCTGGCTGATTGGTGGCTGCATAAGCACCCCGGT<br>TGGCAGTTAAACGTTGCGCGCCGCGGAACCAAGGAAATCGCCTACAC<br>CAACGACGATCTCCCATTCCCGAATTTGGCGACTATCGACCGGCGCGC<br>GCTCAACCGCAAGAAGGGAATGAGCAGCCCCGATCGTTTCCACATCG<br>TGGAGTGTAAGACCGCGATGACGCTGGATGATTGGGGGCGCCCGGGG<br>GAGGAAAACGCGGTGCCGGCCGACTATTTCACCCAGGTGCTCTATCA<br>GATGGGTGTGAGCGGTATTCACCAGGCGTCGATTGTGGTGCTTGGCCC<br>ATTCTCGGAGCCGGAGATTCACGAGATTGAATGGCGCGAGGATCTCT<br>TCGCCGCGCTGGTTGACCAGCTGGCAGAAATCTATGTGTCCACCAAG<br>ACGGGCGAAGTGCCCGATCTTGATGACTCGCTGTCTACCTATGAGACG<br>GTGCGCGGCCTGCACCCGGATATTGACCCGCAGGCTGAAGTTCAGCT<br>CGACCACGCGCAGGCGGTGGCTTGGCTCGACGCCATCGTGGGCGCCG<br>ATGAAGCTGTACGCGCCGCCCAGCTGGCAAAGACCGAACTGGCGGAG<br>CTCATGGGCAATGCGAAGTATGCCCGCGTGGGCACAACCAAGATTGC<br>CGACAGGCGCTCGCGCAGCGGAAAACCGCCCTATGTCCAGTTCAACA<br>AGAAAGCCGACCTCGCGGTCTAA	MTSRILANPPAPGTAEW<br>AKIVTASKAPALLGVSR<br>FTSQYAAWHQLAGLID<br>VDMMDPDRAAWGHIA<br>EASLADWWLHKHPGW<br>QLNVARRGTKEIAYTN<br>DDLPFPNLATIDRRALN<br>RKKGMSSPDRFHIVECK<br>TAMTLDDWGRPGEEN<br>AVPADYFTQVLYQMG<br>VSGIHQASIVVLGPFSEP<br>EIHEIEWREDLFAALVD<br>QLAEIYVSTKTGEVPDL<br>DDSLSTYETVRGLHPDI<br>DPQAEVQLDHAQAVA<br>WLDAIVGADEAVRAAQ<br>LAKTELAELMGNAKYA<br>RVGTTKIADRRSRSGKP<br>PYVQFNKKADLAV
CSP1_12	+	6586	7200	*orf12*	hypothetical protein	ATGCCGAATTGGATTACTAACCGGATCCGTGTGAACCCATCACATTTC<br>GCCGAAGTCAAGGAAATACTGCTCGACGACGCGGGGGAGGTTGATTT<br>CAATCGCCTAATCCCAATGCCCCCATGGATGGATACCCTCAGTATCTC<br>GATGATGGAGCGGGAGAACCCGCGTAAATGGTTTGAATGGTCGCGCG<br>CGCACTGGGGCACTAAATGGAACGCCAAGCACACCGTCGTTAAAGAC<br>GAGCTGTGCGAGATTGCGTTTGATACCGCATGGCAGCACCCGGAACC<br>CGTTATTGAGGAGCTTTCCCGCCTGCTTCCGCACGTGACCTTCGCGGT<br>CATCTACGCCGAAGAAGATCTCGGATACGGGCAGCGCGGGGCTTACT<br>ACATCCGGAACCTAGAAAAGGTCCCATCCCCGGCCGCACTCATGGCT<br>GACGATGGCGTCTGGGTTGCGAACTGGGACTGCCTCGTCATGCGGGG<br>CGAACCGTTGGAATATAGCGAGGATTTCCCAGCCTCAGAGAATGATT<br>CCCGTGATTATGACCCCTTCGATATTCTCCCCGGCTTTGCTCACCTAAT<br>CGGTGACGAGCCTACTTTTAACCCTTTTTGGCTTCACGCCAACTAA	MPNWITNRIRVNPSHFA<br>EVKEILLDDAGEVDFNR<br>LIPMPPWMDTLSISMME<br>RENPRKWFEWSRAHW<br>GTKWNAKHTVVKDEL<br>CEIAFDTAWQHPEPVIE<br>ELSRLLPHVTFAVIYAE<br>EDLGYGQRGAYYIRNL<br>EKVPSPAALMADDGV<br>WVANWDCLVMRGEPL<br>EYSEDFPASENDSRDYD<br>PFDILPGFAHLIGDEPTF<br>NPFWLHAN
CSP1_13	+	7219	8157	*orf13*	RecT-like ssDNA annealing protein	ATGTCTGAAATTGCTACCTACGAGCCAACTAGGGCCGAAATCCAAAC<br>CGCAAAGACCGACTTCGCTAACGCGTCGATTGATGCGATTGACCGCG<br>AACTGGAAGTCATCGAAAAGGCCGCCCGCGTGGTACCAACCTTGATT<br>AAGGGAGGCATGGTGCCAGACATTTACCTGCCTACGCACCAGCCGAA<br>AAAGAACGGCCCCGCCCAGGGCGAGGACGTCGCAATCGCTAAGGCG<br>ATGGCCGCCGCCGTATTCGGCGCGTCGCTCGGATTCGGCATCGCCAAA<br>TCCTTGCAGAATGTGTTCACGGTTCATGGCCAGCCGTCGATTTATGCG<br>CGCACCGCGGTTGCTCTCGTGCAGTCCCGCGGGCATGAAGTATGGGT<br>AGGCGAGGAGACCACCAATTCGGTGACAGTCTACGGGCGTCGCCGAG<br>GCTCGGATAAAGTCTTTGATTCCACCTGGGACATTGAGCGCGCGAAG<br>ACCGCCGGGTTTGTCAGCAACCCCAAGTACAAGTCGCAGCCGCAGGA<br>AATGCTCTACGCCAAGGCTGCGATGGCGGTATGCCGCCGCATGGCCG<br>CCGATGTACTCGAGGGTGTGCCGATGTCGGTTGAGGAGCTGCAGCTC<br>GAAAATCGGCCGGCAACTGTGAAGGCGAGAGCCACTCGCATGGATCG<br>TCCAGGCCGCGGAGCCGATGGGCTACAGGCCGCACTCGGCCGCTCTA<br>CGGCTCGTGAAGAGGCTACGGAGGAGCGGCCTGCTGAAATTGAATCC<br>CAGCCTGCTACTTCGCCGGAGGATGATCAACTACTCGCAGATTTGAAG<br>TCCACGCTGGCAGGCTTTACCACGACCAAACAGGTAAACGCTTTCGTA<br>GCGGAGTTGAAGCAAGACCCGTCCGTTCCGGATGAGGCTCTACAGAT<br>GGCCCGCGACCGCTGGAACGAAATCGCCGGCGGTGAACAATGA	MSEIATYEPTRAEIQTA<br>KTDFANASIDAIDRELE<br>VIEKAARVVPTLIKGGM<br>VPDIYLPTHQPKKNGPA<br>QGEDVAIAKAMAAAVF<br>GASLGFGIAKSLQNVFT<br>VHGQPSIYARTAVALV<br>QSRGHEVWVGEETTNS<br>VTVYGRRRGSDKVFDS<br>TWDIERAKTAGFVSNP<br>KYKSQPQEMLYAKAA<br>MAVCRRMAADVLEGV<br>PMSVEELQLENRPATV<br>KARATRMDRPGRGAD<br>GLQAALGRSTAREEAT<br>EERPAEIESQPATSPEDD<br>QLLADLKSTLAGFTTTK<br>QVNAFVAELKQDPSVP<br>DEALQMARDRWNEIAG<br>GEQ
CSP1_14	+	8154	8588	*orf14*	single-stranded DNA-binding protein	ATGATTGATTACGAGTACCGCTCTGGGCATCTCGCAGCGGATCCGGA<br>GCTGCGCTATACGCCGTCTGGCACACCGGTTGTGAATTTTACGCTGAT<br>TCAATCCTCGAAGTACTACGACCAGCAGCAGGCAACGTGGGTGACCT<br>CCGCGCGCTCTGTCGTGGACGTGGTGTTGTGGGATAAGAAGTTCGAC<br>AACGGCGACGTTATCCCGTGGACCGCCTGGGCAACCGAGGTGCTGCG<br>TAAGGGCTCGCACGTGGTGGTTCATGGCCGCCTGGTTCAGCGCTCGTG<br>GCAGAACCAGCAGGGCGAGAATCGGTACAAGACGGAGTTCCGTGCG<br>GAGAGTGTCTTTGCCTCGCTCGCGGAGGTTGCAACCTCGGACGCGCCA<br>CCGAATGCCGGCATGGGCGGTGGGGCTGCCAGTGCCCCTGGCGCCCC<br>GCCATTCTAG	MIDYEYRSGHLAADPE<br>LRYTPSGTPVVNFTLIQ<br>SSKYYDQQQATWVTSA<br>RSVVDVVLWDKKFDN<br>GDVIPWTAWATEVLRK<br>GSHVVVHGRLVQRSW<br>QNQQGENRYKTEFRAE<br>SVFASLAEVATSDAPPN<br>AGMGGGAASAPGAPPF
CSP1_15	+	8599	8772	*orf15*	hypothetical protein	ATGGCAAATCCTGACGCCCTGGCGGCTTTAACTCGCCTTTCTCGTGAG<br>GCTACCGCCGGTGTATCCGCCGAGGAGATACTGGCTTTTGCCGACGAT<br>AACGGCGTTGAGCTGACCGCAGCGCAGCGTGCACGGCTTCTCCGAAT<br>GTTCCCCACAGAATCGCCCGATTCGGCCTGA	MANPDALAALTRLSRE<br>ATAGVSAEEILAFADDN<br>GVELTAAQRARLLRMF<br>PTESPDSA
CSP1_16	+	8848	9117	*orf16*	hypothetical protein	ATGACTGCCGGTGACCTCAACCCAAAGGTTAAGAACCCAAATTCCGT<br>TAATGAATGCCGCCGGACTATCCCGCGAGGACTTCGCACCATGCTTGC<br>AAGCAAACGCCCGCTGGATGATATGTCCGATGCCGCACTTCGCTGGCT<br>ACAACGCCACGACCTCATCCGCCCCAACAAGCGGGCGGGAGAACCCG<br>GGCAGAGCACCTGGACGTACACCACGACTGGAAGACGCCTCGAAGGC<br>GAACTCGTTAAAGAAGCTACGCGCGCGGCATGA	MTAGDLNPKVKNPNSV<br>NECRRTIPRGLRTMLAS<br>KRPLDDMSDAALRWL<br>QRHDLIRPNKRAGEPG<br>QSTWTYTTTGRRLEGE<br>LVKEATRAA
CSP1_17	+	9283	10059	*orf17*	hypothetical protein	GTGGAGAACCTTTCGAATGCGGCCATTGGGCTATGGGTGAAGTCTGG<br>TTCCTGGTGTTCGAAGCATGAGACGGATGGGGTGATTCCGGCGTCTCG<br>CGTGAAGGCGTTGAAGGGCACTCCTAGTCAGATTCGTGCGCTAATTG<br>AGTGTGGTTTATGGGTCGAAACACGATCCGATTTGGGTGCGAAATGCT<br>ACTCATTTCGATCATGGTTTGACTACCAACCGAGTCGCGAAGAGCGTG<br>AGGAAGAGCGCGCAGTTTGGCGCGAAAAGAAACGCAAATCGCGCCG<br>CCAAAAACTCGACAACCAGCGCAATCGCCAAAATGTCCCCGGGGGAG<br>TCCCCGGAATGTCTACCGAGGGGACATTTGAGGGAGTCACCGAAATG<br>TCCGGTACCATACCCGTCCCGTCCCGACCCGACCCGACCAATATAGGG<br>GGTACAGGTAGCCAGCCAACTTACGGTGATCAAGGGGATAGGCAAGT<br>GGCTGGCAGCCTTACTCTCGATGGGCTAGCGGACGCTACGCGCCGCG<br>CCCGCGACGCCGGCATCCCCGAATCGGCCATCGCCGCCGGGACACGA	MENLSNAAIGLWVKSG<br>SWCSKHETDGVIPASRV<br>KALKGTPSQIRALIECG<br>LWVETRSDLGAKCYSF<br>RSWFDYQPSREEREEER<br>AVWREKKRKSRRQKL<br>DNQRNRQNVPGGVPG<br>MSTEGTFEGVTEMSGTI<br>PVPSRPDPTNIGGTGSQ<br>PTYGDQGDRQVAGSLT<br>LDGLADATRRARDAGI<br>PESAIAAGTREFNRRPE

						GAATTCAACCGACGCCCCGAGCCCAAAGGCCCCGGACTTCTTCGCAC<br>ACTCATCAACGACGCCGCCAAAGCCGAACACTCCACCCAAGCCGACG<br>AAAAGAAGCAACGCCGCGCCGCCATCGACGCGTGCAACCTCTGCGAC<br>GACAACGGCATCCGCTACGCCAACGGCCAAGCCCACCGCTGCACCCA<br>CAAACCCGAACCGCCCTTCTGA	PKGPGLLRTLINDAAKA<br>EHSTQADEKKQRRAAI<br>DACNLCDDNGIRYANG<br>QAHRCTHKPEPPF
CSP1_18	+	10071	10313	*orf18*	hypothetical protein	ATGCGACGCCTCACCAAGCCACGATTCCTCGTGCGATCCACAATTCGC<br>AACGACGGAGTTCGGAGATGGCAAATCCTAGAGCGCACAGGCCTGAT<br>CACCCTGCCATACGGATTTCCCTTCAGCCGCCAGTTCAGAACCTGGCC<br>CGAAGCCCTCCACGCCGCAAACCACCTCGCCCTCAACAACGACCCAT<br>ACCTGAAAGGACTCGAATGCCTACCACCTTTCCGAGCCTCATCGAACG<br>TCTAG	MRRLTKPRFLVRSTIRN<br>DGVRRWQILERTGLITL<br>PYGFPFSRQFRTWPEAL<br>HAANHLALNNDPYLKG<br>LECLPPFRASSNV
CSP1_19	+	10277	10459	*orf19*	hypothetical protein	ATGCCTACCACCTTTCCGAGCCTCATCGAACGTCTAGTCACCCGCGCC<br>GAACACGGCCAACTCACCGAAACCCAACACACCATCGTGGGCCGCGC<br>ATGCGCCCGCCAACTAGGCGCCATCCCCACCCGCAAACACACCCGAC<br>ACCAACCATCCAACCCACGCCCCGTATTCCGCTACCGCTAA	MPTTFPSLIERLVTRAE<br>HGQLTETQHTIVGRAC<br>ARQLGAIPTRKHTRHQP<br>SNPRPVFRYR
CSP1_20	+	10470	10868	*orf20*	hypothetical protein	ATGCACTTACCAAAAATCACCATCAGCCCCAACGCCTTCCGCGTCAAC<br>GGCCAGGAACTCATCGTGATGCACGATCCAGAGCCGACAATCAACCA<br>CATCGGCAGCAACATCTACGGCGTAACCGTCACCATCCCCTCGGACG<br>ACATCGACCTCGACGCTGACGCCCGCTACACCGACGAAACCGACAGC<br>GACGTTGTCGACTGCGAAATCATCACCAACCCACTTCACACCCCAGCC<br>GGAACCCCATTCCTCGCCACCGTCTGCGGCGGCCTCCCACAACTCGTC<br>GTCCGCAGCCACAGCGACATCGATCTAGAAGCCTGGTGGCTCCTCAA<br>CGGCACCCGCTGGCTACGAACCGACGATTGCGACGTCGAAATCATCA<br>CCCCACTCAAATCGGACTAA	MHLPKITISPNAFRVNG<br>QELIVMHDPEPTINHIGS<br>NIYGVTVTIPSDDIDLD<br>ADARYTDETDSDVVDC<br>EIITNPLHTPAGTPFLAT<br>VCGGLPQLVVRSHSDID<br>LEAWWLLNGTRWLRT<br>DDCDVEIITPLKSD
CSP1_21	+	10900	11067	*orf21*	hypothetical protein	ATGACTAACCATTACTGGCCGGGGGCTGACAGCGTCCGCGCCGCCCG<br>CCTCGACTTCGATCCAGATGATGACGGGTTCGACTACATCACCTTTCG<br>CGTCCCGACCGGGTACACGTACAACACCGCTCAGCGCTACAAAATCG<br>AACCGCTAGGAAACCACAATGACTAA	MTNHYWPGADSVRAA<br>RLDFDPDDDGFDYITFR<br>VPTGYTYNTAQRYKIEP<br>LGNHND
CSP1_22	+	11060	11470	*orf22*	hypothetical protein	ATGACTAACACCATCGAAGTACCAATCTCACTAATCAACGCCGGAGA<br>CATCGACGCAATCCGCGACCTGCTACCGCAGGAAAACCTTTTTGGCA<br>GGTGGGCAGAGCACCCCACGCTTGGGCGCGGAATCATCATCAGCGAA<br>CATCCAGACCAAGAGAATTTTGTGAAATTCGTCAATGGTAAATCGTG<br>GTCGGGCGTAATCCTCGACGACCTCACCCTTGACCCGGTAGAACTCGT<br>AGCACTTAAGGACTTCGAGGGCGCGCCAGAGGGCACAGTCATTTCCG<br>ACACGGGAGAAAATGCCTACCAAAAACTCATCACCGACGCGTGGGAA<br>AGCAGAAATGACTACCTCACCGCTAAAGAAATGGCAGTCAGCGGCCC<br>GTGGAAAATCCTCCGCTGGGGATGGGGAGAATAA	MTNTIEVPISLINAGDID<br>AIRDLLPQENLFGRWAE<br>HPTLGRGIIISEHPDQEN<br>FVKFVNGKSWSGVILD<br>DLTLDPVELVALKDFE<br>GAPEGTVISDTGENAYQ<br>KLITDAWESRNDYLTA<br>KEMAVSGPWKILRWG<br>WGE
CSP1_23	+	11473	11811	*orf23*	hypothetical protein	ATGCCTAAGACCCTCGCAGACATGACCCCAGAGCAGCGCGCCAACTG<br>TGTCGGAATGTGGTGCAATATTGGGCCGCATGAGCCGGGCATTGACG<br>CGGGAAGCTACCTTTTCATCCTGCGCTCAAGCAGCAATTATCGAGGTG<br>CTGAGCATGCAAAACTTCTTGACCCAATTAGCGGGGTGACTGAATGC<br>ATCTCATTCAAGAACGTCACCCCACGCCCTGACCTCCCCCGCGCATGG<br>GCACTCGACGGCACACCACCAGCAGGGGAATGGGAGCACACCCACAT<br>CCCCGCCCTAGGAGAGTCCACACGCCGATGGATAGGAGACTGGGAGC<br>AAGCATGA	MPKTLADMTPEQRANC<br>VGMWCNIGPHEPGIDA<br>GSYLFILRSSSNYRGAE<br>HAKLLDPISGVTECISFK<br>NVTPRPDLPRAWALDG<br>TPPAGEWEHTHIPALGE<br>STRRWIGDWEQA
CSP1_24	+	11808	12047	*orf24*	hypothetical protein	ATGAGCCGTGTCATCCCCGCCTACGTGCTTCAAGCCAAGCAACGCAA<br>GGCCAGAGCATCGGAACGCTTCGAGGAAGAAAACCAACGCCTTGAAG<br>CCCACACGCGAGCCACCAAGTGTTGCACGCTCATAACCACCGGCGCG<br>CACCCAGAAGACACCCACCTATGCCTCCGCAAAAAGGGCCACAAGGG<br>CTACCACCAAGACAAAAACACCGGACTCAGCTGGAAATGGGAGGAA<br>GCATGA	MSRVIPAYVLQAKQRK<br>ARASERFEEENQRLEAH<br>TRATKCCTLITTGAHPE<br>DTHLCLRKKGHKGYHQ<br>DKNTGLSWKWEEA
CSP1_25	+	12044	12223	*orf25*	hypothetical protein	ATGACCCCAGGGCAAGCACGCCACCTAATCAACGCGGATAGCCGTAG<br>CACACACGGCCCACTTACCGACTGCCGATTCTGCGAATTGGTAAAGC<br>GGCGAGCGTGGGAGATGGTACACCAAATCGCAAACATGACCTACGAA<br>TACGCCGAGCAAGCCCAACCCGAAGAGGTACAGCCATGA	MTPGQARHLINADSRST<br>HGPLTDCRFCELVKRR<br>AWEMVHQIANMTYEY<br>AEQAQPEEVQP
CSP1_26	+	12220	12423	*orf26*	hypothetical protein	ATGAATTACCAGACCGCGTTTGAAATGCTTCGCGAAATGGTGCGCAC<br>CGAGTATGAGAAAGCTAGCCGCGAATTCAACAGGCACCACCCCTCAC<br>CACTCGTCGCGGCGGAATCCGGCGAGTACCAGCGCGGCAAGCTCAAC<br>GCCGCAGCCGACATTTACAACGAATCCAACCGACTACTACGCCACAT<br>CAAGGGGGAGTCATGA	MNYQTAFEMLREMVR<br>TEYEKASREFNRHHPSP<br>LVAAESGEYQRGKLNA<br>AADIYNESNRLLRHIKG<br>ES
CSP1_27	+	12420	12764	*orf27*	hypothetical protein	ATGAGCAAATGGAAAGTTATGGGCGGCAGATTACGCGGTTCTACTTT<br>CTGGGAGGTGTTCAACCACAACGGCAGATTCATGGAGTCATTCACCA<br>CCCACGCCGAAGCCCTGGCCTACGCAGACCGCAGGGCAAGAACACGA<br>GAATACGTTCTACCTCGCCAGCCACTACCGCTATCACTACCGGGGCTA<br>GAGGACGGCGACGCGCCGATAGTTGCCACATACGACAAAGACGATTG<br>CGTCTACCTCAAGGATGAGGATCACAGCGAGACGCTAACGCTTTACG<br>CACGCGAACTCCGCCCCCTCGCCCTAGCACTACTCGCACACACAGAA<br>AGGATAGAGAAATGA	MSKWKVMGGRLRGST<br>FWEVFNHNGRFMESFT<br>THAEALAYADRRARTR<br>EYVLPRQPLPLSLPGLE<br>DGDAPIVATYDKDDCV<br>YLKDEDHSETLTLYAR<br>ELRPLALALLAHTERIE<br>K
CSP1_28	+	12761	13105	*orf28*	hypothetical protein	ATGACCAACATCGACAAAGCCGCTGACCTGCTTTATCAACGCTCAAA<br>AGAACTACTAGGGGTAGACAGCGATGCCGCACACAGAGCCTGCCAAG<br>AACACGCCCAAGCCCTCGCAGACGCAGGACTACTCACACCAGAACTG<br>CCGGAACCGAACGACCCAGGCATTTTCGTACCAGACGGCAAAGGATG<br>GATACCCGGAGGGCCACACGGGCCTAGCGTGTGGACAGCACCAGGCA<br>GCCCAATCATGGTGCAACGAATCGAACCCGGCGACCTCACACCCGAC<br>GAAGCCCGAGGTGTCGCCCACGCCCTACTCGCAGCAGCCAACCACGC<br>GGAGGAGCAAGCATGA	MTNIDKAADLLYQRSK<br>ELLGVDSDAAHRACQE<br>HAQALADAGLLTPELP<br>EPNDPGIFVPDGKGWIP<br>GGPHGPSVWTAPGSPI<br>MVQRIEPGDLTPDEAR<br>GVAHALLAAANHAEEQ<br>A
CSP1_29	+	13102	13470	*orf29*	hypothetical protein	ATGACTATTCAAGGATTCCGCATAACCAACAAGGGCCGCGCAGCCCT<br>GATAGCGATGAAGGAAAATGCGAAGCAGGATGAAGAAGTCCCGGCT<br>CACTACCGCATAGCAGATGCCCTCGCTGAGGCGGGCCTACTCGCGCCT<br>GACCTGCCCCGCGCCTGGGCACCAAACGGCAAACCCGCAGCAGGGGA<br>GTGGGAATACGCGGTGCAATACCTGACCCCGGACGGCTGGAAATACA<br>GCCGCGAATCGTGGGATAACCGTTGGCAGGAAAGCGAAGCCGTCCAA<br>GAAGTCCGCGCCCACCGCGACCACCCCGACGAAGAAACACGCCTCGT<br>CCGTCGCCTCGTTTCCGAATCCGAGGTGATGGAGGAATGA	MTIQGFRITNKGRAALI<br>AMKENAKQDEEVPAH<br>YRIADALAEAGLLAPDL<br>PRAWAPNGKPAAGEW<br>EYAVQYLTPDGWKYSR<br>ESWDNRWQESEAVQE<br>VRAHRDHPDEETRLVR<br>RLVSESEVMEE
CSP1_30	+	13467	13865	*orf30*	RusA family crossover junction endodeoxyribonuclease	ATGAGACTGCACTTCGAAATCCCAGGCCGCCCCGCCGCCCAAGGCTC<br>TAAACGCCACGTAGGCGGAGGCCGCATGATTGAGCAATCCAAATACC<br>TCAAACCCTGGCGCGACAAAATGATCACCACCATGCGCCACCAATAC<br>ACAGGACAACCGCTAGATGAACCCATCGAGGTCACAGCGGTATTTTT<br>TATGCCCAAACCCAAACGCCCTAAATTCCCCGAACCAGCCACACCCC<br>CAGATGCCGACAAACTATGCCGCGCACTAGGCGACGCCCTCACAATG<br>GCCGGCGTAATCAAAGACGACGCCCGCATCACCACATGGCACGCCAC<br>CAAAAAATATCACCCACAAGGCTGGACCGGTGCACACATCACCATCA<br>AGGAGGACCCATGCCACCAGTAG	MRLHFEIPGRPAAQGSK<br>RHVGGGRMIEQSKYLK<br>PWRDKMITTMRHQYTG<br>QPLDEPIEVTAVFFMPK<br>PKRPKFPEPATPPDADK<br>LCRALGDALTMAGVIK<br>DDARITTWHATKKYHP<br>QGWTGAHITIKEDPCH<br>Q
CSP1_31	+	13853	14437	*orf31*	hypothetical protein	ATGCCACCAGTAGATGAACGCGACCTACGAGACCTAGCCCGCCACCT<br>CGCCAACCTCTACCGCGAGCTCGACAGCCTCAAGTACGCACGACCAA<br>CCCCGCCCGAAGTCCGCGCCATGAAACCCGCACCCGGGCCACAATCC<br>CCCGGCAACTGGCTCTACGTCTCATGCTGGCTCGACCAAGAGCCCCGC<br>CTGCGCGAAGTAGCCTTCAACGCCTTCAGCGATATCGGGGTCAAAGT<br>CCGGGACGACGAAGCTGGGGCAGTCGCACTCTGCCAAAAACTAGCCT<br>TCCACGCACAGGCAGTGTCAGAACTCGACTGGGCAAGCGACCTCACA<br>GACGAACTCGAACACCAGACCCGAATCCTTGACAGGAGATGCAACCC<br>ACCGCAACCTGCAATCATCGCCAACCAACCCGAACCACGACACGGAG<br>CCGAACGCATCGCACGACAACTCAGAGCACGCGGCATCCCCACCACC<br>GCCGACACCATCCGAGGATGGGCACGCTCAGGACACATCACCCAAAC<br>CACCATGCCCAACGGGCGCAACGGCTACCTACTCACCGAAGCCCTCA	MPPVDERDLRDLARHL<br>ANLYRELDSLKYARPTP<br>PEVRAMKPAPGPQSPG<br>NWLYVSCWLDQEPRLR<br>EVAFNAFSDIGVKVRD<br>DEAGAVALCQKLAFHA<br>QAVSELDWASDLTDEL<br>EHQTRILDRRCNPPQPA<br>IIANQPEPRHGAERIARQ<br>LRARGIPTTADTIRGWA<br>RSGHITQTTMPNGRNG<br>YLLTEALNHARHR

						ACCACGCCCGCCACCGCTAG
CSP1_32	+	14528	14812	*orf32*	HNH endonuclease	ATGGGCTGGGCAGACTCACGTCACCAAAAGAAACTCGCTGCAGACTTCAAAGCACAGTGTGCCCGTAACGCCACACCGTGCCACCTATGCGGCCAACCCATCGACTACACCGCCCCGCCGCAAACCCCGGACGCCTACGAGCTCGACCACTACTACCCACGCAGCACACACCCCGAGCTCACCGACGACCCCGCTAACTTCCGCGCCGCGCACTCCAACTGCAACCGCAGCCGCGGCAAAGGAACCGCCGTCTACGCACTCGGACAGCAATCAGAACAATGGTGA
						MGWADSRHQKKLAADFKAQCARNATPCHLCGQPIDYTAPPQTPDAYELDHYYPRSTHPELTDDPANFRAAHSNCNRSRGKGTAVYALGQQSEQW
CSP1_33	+	14931	15254	*orf33*	hypothetical protein	TTGGCTTTAAGGAAGAGCTATCCGACCGACGACGATCTCGAGGACGTCGAAATCAGCGAGTTTCCGTGCATGCGGGATGCCGTTCGCAAGTCTGTAGAGACGGCGGACCATCTCGAGGACACTGACGAGGCTGACATTCAGCTCGCATATCAACTTGCGGACATTATCGATGATGCGCGGGAGTCGGGCGACCCGGACGCGATTCACAAGACTGCGTTTGGGCCCATGCCAACTTTGCATAAGGTGTTGACGTCACTCGGGTTAAATCCTGAAGGGCGCGACAAGCTTGGGCTGAATACTCAAGAGGATGACGAGGACTGGTAA
						MALRKSYPTDDDLEDVEISEFPCMRDAVRKSVETADHLEDTDEADIQLAYQLADIIDDARESGDPDAIHKTAFGPMPTLHKVLTSLGLNPEGRDKLGLNTQEDDEDW
CSP1_34	+	15258	16775	*orf34*	terminase large subunit	ATGGCGCCTCTGGTTGGTTCCACGGTGCCGCGTGTTTTCACTCCGCCGCTGCGCGAGCTGACCCCAGAAACGTCGTGGGGCTTTGATGTTGTGTGGTTTGCGTGCGAGATTTTGCGCCAGCCGTTGTCACCGTGGCAAGAGTGGCTGGCAATTCACGGCCTTGAGCTCATGGACCAGGACAAGGTCAAGGAACTTTACCCCGATGACCCCGCCGTGTGGGACGAGGAAATCCCAAGGTTCAAAACAATTTTGGTGCTTGTGGCTCGCCAAAACGGAAAGACGCACTTCGCGAAGACGCTCATCAAGTGGGCACTGTTCCGAAAGCGCCTGAAATACATCCTCGGCGCAGCTCAGACGAAGAATGATGCTTACGAGTTGTGGGAAGACATCGAGAAAGAATGCGATGAGAACCCACGGCTGAAGAAGCGCATGAAGCGCACATCCTTTGCGCACGGCTTCGAGGCGCTGCGTTCTAACTGGGGTGTGTACCGCATTGCGGGGCTGGACCGTAAGGCAGGCCGCGGTAAGACGGCGAACCTGCTTTACATGGATGAGCTGCGTGAGCATAAGGACTGGGCTGGATGGTCGGCACTGTCATCGACGACGAACTCGCCGTTGGTCGGCTTCAATGTGGCTACCTCGAACGCTGGTGATGCTCGCTCCGTAGTCCTGCGGCACCTGCGCGATACCGCCACGAAGGCGATTAATGAGCAGCGCACTGAGACGGCGACGCTGTTTATGGCTGAGTGGTCCGCGGATCCTGATCTCGACCCTGGCGATAGGCAGGGCTGGGCGCAGGCCAACCCAGACCTAGGTAGTAGCCGATTGACGATCCGTGATATTCAAGCCGAGTTTGATACCAAGACGGAGGCGGAATTCCGTACTGAGAACCTGTGCCAGTGGGTTGATGTGCTCGCCCCGTCGAAGTTCCCTGAAGGATCGTGGGCATCATGCCTAGACCCGGAGTCTCGGCGCGCGCCGGGGGAGAAGGTGTTTGTTGGTTTGGACGTGGCGGTGGATTTGAAGGCTGCGCATGTTGTCGGTGCGATGAAGCGTGCGGACGGTGCGTGGCACATTGAGTTGTTGGCTTCGCGTCCGGGCATTGACTGGGTGACTGGCTGGTTCGAGCAACGTCGTGAGAGCGGGGAGTGGTTCGACGGGGAGGTTGCTGTCCAGGCGCGTGGCGCTCCGGTGGGCACACTTATTCCGCAGCTGCGAGAGGCGGGATTGACGGTGCGTGAGTGGGCTGGGCCTGACCTGACTCAGGGAACCCTAGCGTTCTATGACTTGGTCGCGCAGTCGAAGTTGCGGCACCGTGGACAGCCCGCGCTTGATGCCGCTGCCACGGGTGCGCAGGACCGGCGTGCTGGCGATGCGTTCATGTGGGACCGGTCTAAATCAGTGGGTGATGTTGCCCCGATTGTGGCCGCGACCGCGGCTGTTTGGCTTGCCTGTCAGCCGGAGGTAGAGCCGCCGAAGCAATCGGCGTACGAGACGGCTGATTTGATGATTCTTTAG
						MAPLVGSTVPRVFTPPLRELTPETSWGFDVVWFACEILRQPLSPWQEWLAIHGLELMDQDKVKELYPDDPAVWDEEIPRFKTILVLVARQNGKTHFAKTLIKWALFRKRLKYILGAAQTKNDAYELWEDIEKECDENPRLKKRMKRTSFAHGFEALRSNWGVYRIAGLDRKAGRGKTANLLYMDELREHKDWAGWSALSSTTNSPLVGFNVATSNAGDARSVVLRHLRDTATKAINEQRTETATLFMAEWSADPDLDPGDRQGWAQANPDLGSSRLTIRDIQAEFDTKTEAEFRTENLCQWVDVLAPSKFPEGSWASCLDPESRRAPGEKVFVGLDVAVDLKAAHVVGAMKRADGAWHIELLASRPGIDWVTGWFEQRRESGEWFDGEVAVQARGAPVGTLIPQLREAGLTVREWAGPDLTQGTLAFYDLVAQSKLRHRGQPALDAAATGAQDRRAGDAFMWDRSKSVGDVAPIVAATAAVWLACQPEVEPPKQSAYETADLMIL
CSP1_35	+	16785	18116	*orf35*	phage portal protein	ATGGGGCTTCGTGAGTGGCTTGGCTTCAGTAAATCGACGATGTTGCCGGAGCCGGGCGAGTTGCAAGCGCCGATTGTGTCGGCGTACATGGATGACCTTCTGATGCAGATTCGGAATCAGTCGCCGGCGGACTTGTGGCGTTCGCAGCCGTATCTTCGGATGGTGATTTCGTTCCGCGCGCGAAACGTGGCTCAGATTGGGCTGCATGCCCTGGAGCTGATGCCGGATGGTGGGCGTGAGCGGGTGCGCTCCGGCCCGATTGGTTCGCTTCTGCGCGAACCGAACCCTGACCAAACATGGTACGAGTTGCTGTTCGGTTTGGTGTCTACGTTTGACCTGTACGATGAGGCGATTCTCTACGTTGCCGACGACGGGGAGAAGATTCTGCTGCGTCATATCCCGAGGAATTGGATTCTTCAGTGGATTGACGCGGATGCCTACGGTGTTCGTGGCGCTCGCGTTCATTTCCCTGGTGATGATGCTCATGGGCGGATTGTTCCTGAGGACAATTTGATTCGTATTCATGGGTGGTCGCCGGAGGATGAGCGTTTCGGGGCGTCGCCGATTGAGGCGCTTCGGGTGATTCTCGAGGAACAAGTTCAGGCGCAGATTTTCCGGCTTCAGATGTGGAAGCGCGGCGGGCGTGTGGGTTCTTATGTGACGCGCCCTAAGGATGCGCCGGATTGGACTCCGGAGGCACGCCAGCGGTTCAAGCACTCTTTCCAGGCGGCGTTTACTGGTGAGAGTGGTTCGCAGGCTGGTGGTGTTCCGGTTTTCGAGGACGGTATGGAGTGGCGAAAGCTGTCCGTTACCGCCCATGAGGAGGAGTTCGTGGAGGCGTCGAAGCTGGCGCTGACCACGGTGGCTGGTGTGTACCACGTCAACCCGACAATGCTCGGCCTGCTCGACAACGCTAACTACTCCAACGTGAAAGAGTTCCGTAAATCGCTGTACGGCGACACGCTGGGGCCGCTTCTGAAGCAGATTAGCGAGAAGCTGAACGCCCGAATCCTGCGTTACGTGTCAGAACGTAGTTATCTCGCTGAGGAGCATCCGAATCTGGAGAATATCTATTTCGAGTTCAATCTTCGGGAGAAGCTTCAGGGAAACTTCGAGGAAGAGGCCGCGGTTCTTGGCGGGGCAGTCGGCGGGCCGTTTATGACGGTCAACGAAGCGCGCTCACGCCAGAACATGCCGGCGGTTCCGGAAGGCGACCGACTCATCCAGCCGCTCAACCTCGGGTTGAGGGGTGAGGGGGAGTCTTCTGGTGACCAGGAGGTGGTTGATCCGCCGGATGACCAATCAGCTTTACCTGAAGGGGAAGAAGAATGA
						MGLREWLGFSKSTMLPEPGELQAPIVSAYMDDLLMQIRNQSPADLWRSQPYLRMVISFRARNVAQIGLHALELMPDGGRERVRSGPIGSLLREPNPDQTWYELLFGLVSTFDLYDEAILYVADDGEKILLRHIPRNWILQWIDADAYGVRGARVHFPGDDAHGRIVPEDNLIRIHGWSPEDERFGASPIEALRVILEEQVQAQIFRLQMWKRGGRVGSYVTRPKDAPDWTPEARQRFKHSFQAAFTGESGSQAGGVPVFEDGMEWRKLSVTAHEEEFVEASKLALTTVAGVYHVNPTMLGLLDNANYSNVKEFRKSLYGDTLGPLLKQISEKLNARILRYVSERSYLAEEHPNLENIYFEFNLREKLQGNFEEEAAVLGGAVGGPFMTVNEARSRQNMPAVPEGDRLIQPLNLGLRGEGESSGDQEVVDPPDDQSALPEGEEE
CSP1_36	+	18113	18895	*orf36*	putative prohead protease	ATGACAGTAAAGAGCGTCATCGCTGAGGTTAAAGCCCTCGGCGATGGAGAAAAGGCCGAGTTTGAGGCGTACGCGTCCGTATTCGGTAACCGTGACAGTTACGGGGACGTAGTGCAGAAGGGCGCTTTTGCAGCGTCGTTGAAGGCATGGGACGAAAAGGGTGCTCCGATTCCGCTGTTGTGGGGCCATAATATGGCCGATCCGGACTTCAATATCGGCATGGTGACCTCCGCTGAGGAGGACGAGCACGGGCTGAAAGTGGTCTGCGAGCTTGATACCGACTCGCCGAAGGGTGCCCAGGTGCACCGCCTACTCAAGCAGGGTCGTGTTCGGGAGATGAGCTTTGCTTTCGCCGCGACGTCTAGTGAGTATGGCGAGCTTGATGGTAAGAGCGTGCGATTCCTCAAGGAAGTTGACCTGTTTGAGGTGTCCGTCGTGCCGCTCGGCGCGAATCCGGAGACCGAAGTGCTGGCGGTGAAATCCCCGACGGTGAACCTAACACTGCCGGAGACGCTTGCCGAACGATTCCTGGAGGTTCTGGGCGGGAAGTCCCTGGGCGGGCCACCGGGAACTGATGAAGAAGATGACGATGAAGATACCCCCGATTCTGAGGATGAGTCCTCGGATGAGGGGGTTTCGTCATCTGAGGTCACTGATAGCGGTGGCGAGGACGAGCCCGGCGAGGATGCCGGTGAATCCGATGAAGACGATGAGGACGAGGAAGGCCGTAAGGCTGACCTTGACCGATTCGAGACCATGTTCCGCGCCGTGGGGCGCGACTAA
						MTVKSVIAEVKALGDGEKAEFEAYASVFGNRDSYGDVVQKGAFAASLKAWDEKGAPIPLLWGHNMADPDFNIGMVTSAEEDEHGLKVVCELDTDSPKGAQVHRLLKQGRVREMSFAFAATSSEYGELDGKSVRFLKEVDLFEVSVVPLGANPETEVLAVKSPTVNLTLPETLAERFLEVLGGKSLGGPPGTDEEDDDEDTPDSEDESSDEGVSSSEVTDSGGEDEPGEDAGESDEDDEDEEGRKADLDRFETMFRAVGRD
CSP1_37	+	18914	20122	*orf37*	major capsid protein	ATGACTGTTGAACTGAAGAAGCGTCGCGCCAAGCTGGCCGACGAGGGCCTTAAGATTGTCACTGAGGCTAAGTCCGCGGGCGCTCCGTCCGCCGAGCAGGTAGCCCGCCTTAAAGACATTGAGTCCGAGCTCGGCACGCTGGACGCTCAGCTTAAGGCAGCGGAGAATTTCGGCTCCTATGAGCAGCTTTTCAAGGGCATGCCGGAGGCCAAGAAGGAAGAAGCCGCCCGTACCCTGGGCGAGCACTTCCTTAAGTCCGCAGGTGACCTGTCTGTCCTGCGTGACCGCTCTAACTCCACTGTTTCCGCGACGGAGTTCAAGGCGAACACGGATTACCGTCACCACCGCTGGTGATGCACTGACTCCGGTTCTGACCGATGTGGACAAGACTGTCGTCCACGGTAAGCGTGACCGTCCGGTCCTGGCTGA
						MTVELKKRRAKLADEGLKIVTEAKSAGAPSAEQVARLKDIESELGTLDAQLKAAENFGSYEQLFKGMPEAKKEEAARTLGEHFLKSAGDLSVLRDRSNSTVSATEFKANTDTVTTAGDALTPVLTDVDKTVVHGKRDRPVLADLLGS

						CCTCCTCGGCTCCGGAACTATCTCCGGCAACGCCATCAGCTACTTCAT<br>TGAGGGTGCGTTCGAGGGTGACTTTGGCACCGTGGCTGAGGGTGCGC<br>AGAAGCCGCAGGTACATGTGGCGAACCCAACCGCGGTCACCGACCCG<br>CTGCGCAAGATTGCCGCATGGATCACCATCACCGACGAGTTCCTCGA<br>GGATCTTCCGTTCCTCAAGTCTGAGATTGATAACCGCCTGCTGTACAA<br>GCTGGCTATCTTCGAGGAAAAGCAGCTCCTGTCCGGCGACGGCATGG<br>GTAACAACATTAAGGGCCTGCTCAACCGCGACGGCCTGCAGGTGGAT<br>ACCGCCGCCTCGATTGATGAGGTGCCGGATAAGATTTTCTCCGCAACC<br>ACGAAGATTTCCACAGCGACCGAGCTCACTGCCGACGGCGTGATGAT<br>CAACCCTGCCGACTACGAGCCAATCCGGCTGAAGAAGGACGACAATG<br>GCCAGTACTACGGTGGCGGGTTCTTCCAGGGTGCCTACGGCAACGGC<br>ACCGTTATGGAGCAGCCGGGCCTATGGGGCCTGCGCACCGTGGTCAC<br>CCCAGCCATCCCGAAGGGCACCGTCCTCGTCGGCGCGTTCGGCCTGG<br>GCGGCACCGTGTACCGCAAGGGCGGTGTGCGCGTGGAGGCAACGAAC<br>ACCCACGCGGATAACTTCACCACCAACAAGGTGACCATCCGCGCTGA<br>GGAGCGCCTCGCATTGGCAGTGCGCTACCCGTCCGCTTTCGTCAAGCT<br>GACCGTAGGCGGTGCTGAGTTCTAA	GTISGNAISYFIEGAFEG<br>DFGTVAEGAQKPQVHV<br>ANPTAVTDPLRKIAAWI<br>TITDEFLEDLPFLKSEID<br>NRLLYKLAIFEEKQLLS<br>GDGMGNNIKGLLNRDG<br>LQVDTAASIDEVPDKIF<br>SATTKISTATELTADGV<br>MINPADYEPIRLKKDDN<br>GQYYGGGFFQGAYGN<br>GTVMEQPGLWGLRTV<br>VTPAIPKGTVLVGAFGL<br>GGTVYRKGGVRVEATN<br>THADNFTTNKVTIRAEE<br>RLALAVRYPSAFVKLT<br>VGGAEF
CSP1_38	+	20122	20301	*orf38*	hypothetical protein	ATGAAGCTCTACAAGCGCATCGGCGAGAACGGCTTTGAGCACATCGT<br>TCAGGCCGAGTCCGACGAGGTAGCGGCCCACATGGGACTGCTGCCTT<br>ATCAGGCAGAGACCAAGGAACGGAAGCCTGCTAACAAGGCTGCTCGT<br>GGAGCAACCAAGGCCGCTAAGCCGGAACCGAAGGAGTGA	MKLYKRIGENGFEHIVQ<br>AESDEVAAHMGLLPYQ<br>AETKERKPANKAARGA<br>TKAAKPEPKE
CSP1_39	+	20307	20867	*orf39*	hypothetical protein	ATGACTGAGTCGCTGGAATCCTCGGAGCTGACGCCTGAGCAGGTCAC<br>AGACCTATCTGGCGGCTTGGTACCTGCCTCCACTCCTGGGCTGGCGGA<br>GAAAATCAGTGCCACGCTCGCCGGCATCCGCAGACTGTGCGGCTGGC<br>ACGTGTTCCCAGTGAAGGAAGAAACAATCACGGTTGATGCGTTCGGT<br>GGGACGTTGCTGCGTCTGCCGACTATGCACGTGGAAGACATTTCCCGT<br>GTTGTTATCCGCGGCGCCGAGGTTGATCCTTCTAGCTTTGGGTGGTCT<br>CAGTCCGGGATGCTCGAGCTCTACGAGGGGGAGTTTCCCGACCGGTTT<br>AGGTCGGTTGAGGTAACCCTGCGCCATGGGTTCGACCAGGCCCCTGA<br>CCTTTTGCGCATTGCTGCCGAAATTGTTCAGCGGTCGGTACTCGCTGG<br>TACAGGCGGCAACGTCAGCGTTGGTGGTATCAGTGTTGGTGCCCCGG<br>CTGGTGGCGGGGGCGGTGGGTCCATCACCCCGATGGCTACTGAGTGG<br>CGCATCATCGACCAGTACAAGCTGCGTGAGTGGCCATGA	MTESLESSELTPEQVTD<br>LSGGLVPASTPGLAEKI<br>SATLAGIRRLCGWHVFP<br>VKEETITVDAFGGTLLR<br>LPTMHVEDISRVVIRGA<br>EVDPSSFGWSQSGMLE<br>LYEGEFPDRFRSVEVTL<br>RHGFDQAPDLLRIAAEI<br>VQRSVLAGTGGNVSVG<br>GISVGAPAGGGGGSIT<br>PMATEWRIIDQYKLRE<br>WP
CSP1_40	+	20864	21229	*orf40*	hypothetical protein	ATGATGCAGCTGATTTTTACCGAGCGGGTTGAGATTCTTCGCCCGCGG<br>CGTATCCGTGGTGACTATGGCGGCTTCGTTGAATCCTGGGATGACCCG<br>GAGGTCATTCCTGTTGCCGCGCCGGTCAGTGTCCAGCAGGTGTCCACG<br>AGTGAGGACGAGACAGGCAGTAGCCAGCTCGTCGTGACGAAGTGGCG<br>CCTGTACTCGCAGCCGCCCCATTTGCTTGACCAGCTCGAAACCAATGA<br>CCGGATTCGGGTTATTGGTGGGGTTGGTGGCACGTGGGTGGTGGGCG<br>ACCCGGAGCACTGGCGCGGGCCACTACTTCCGCACACTGAGGTTGAT<br>TTGGAGGTCTACCGTGGTACGAGTCGAGCTTGA	MMQLIFTERVEILRPRRI<br>RGDYGGFVESWDDPEV<br>IPVAAPVSVQQVSTSED<br>ETGSSQLVVTKWRLYS<br>QPPHLLDQLETNDRIRV<br>IGGVGGTWVVGDPEH<br>WRGPLLPHTEVDLEVY<br>RGTSRA
CSP1_41	+	21210	21482	*orf41*	hypothetical protein	GTGGTACGAGTCGAGCTTGATACTGACGCGATTTTCCAGCAGGTGATG<br>AACGCCAACGCGGTCCATGCGAAAGTGCACAATCGTGCGGCCAAAAT<br>CTCTACCAAGATTCGCCGTGACCTGAACAAGGCCGGGATTGATGCCG<br>GCGTGGAGGTCAAAGAGTACGCGCACGCGAATGGCCGCTTTGGCCTA<br>AACATCGTTGGACACGTTGACGACAAGGACGCTCGGCGTGCTGGACG<br>CATTGCCCGCCGTGCCGGAAGGAGTGTACGCCGATGA	MVRVELDTDAIFQQVM<br>NANAVHAKVHNRAAKI<br>STKIRRDLNKAGIDAGV<br>EVKEYAHANGRFGLNI<br>VGHVDDKDARRAGRIA<br>RRAGRSVRR
CSP1_42	+	21479	21883	*orf42*	hypothetical protein	ATGAACGACGTTGACATACTCAAGTTCGCCATTGATGCGGTGGCGGA<br>GGTTCTCGGAGATGAGTTGTGGGTGGCGGATCATCTGCCGCCCGCGG<br>ACGAACTCGAGAAACTGACCCCCGCGGTAACAGTTGACCTCCTCCCC<br>GGCAGTGAAGTCGTGCCGTGGGGAGCGGACACGGGCGAGTACATCTC<br>GGAGATTATTTCTCTTGATGTGGAAGTCGTCGGTGTGTCTCGCGCTCA<br>GTGCACGCCGGTAGGGGACAGGGTGCGCGCCGCTCTGCACCAGCTGC<br>CGTTCCTAGAGGGCAGCGGTGTGAAAAGCGTGGACTGTCCGCGTTTTT<br>CTACGCGTGAGGATATTAACCCGCAGCTCCGGGTCTTGGGGGCTGTA<br>GTCGATTTGATGGTGCTCAAGACCTAG	MNDVDILKFAIDAVAE<br>VLGDELWVADHLPPAD<br>ELEKLTPAVTVDLLPGS<br>EVVPWGADTGEYISEIIS<br>LDVEVVGVSRAQCTPV<br>GDRVRAALHQLPFLEG<br>SGVKSVDCPRFSTREDI<br>NPQLRVLGAVVDLMVL<br>KT
CSP1_43	+	21975	22892	*orf43*	Ig domain-containing protein	ATGGCAGACCAGAAGACCCTGGAGGGCTTTAACGCCCGCAAGGCCCG<br>CGTTGGCATGACCGGCGCTGTCCGCTCCTCCGCCCTCGGCACCGAACC<br>GGTCCCGTTCGGCGAAAAGTACAACACCGATACCCATTACAACCTTG<br>GCTACATCTCCCCGGATGGTCTGGAGATTAGCTTCGATGAGGATAAGC<br>AGGAATACATTCCTTGGCAGGAAGTTTCCGCAATCCGCACCGACATC<br>ACCAAGGCTGTGAAGTCCGTCAAGCTGACCCTGTGGGAGACCGGCAT<br>TGAGAACTTCGCGAAGTTCCTGGGTGTGTCCGAGGATGCGCTGGAGG<br>ACCAGGGTGACGGCTCCTTCGCCTTCTACGAGGACGCGCTGCCGCAGT<br>TCGGCCACGAGCACCTTCACATTGATGTTGTCGATGGTGACAAGGCTC<br>TGCGCCTAGACCTGTTGGATGCGCAGATTACTGAGCGTGGCTCGATGG<br>TCTTCAAGAAGGACGAGATGTTCGGCCTTGAGGTTACCTACACCTCCT<br>ACCCGGCATCCTACGAGGATTACAACGCCACCCTTCCGGAGGGTGTG<br>GGTAAGACTGCTCGTTGGCAGATGAACTCCGCTTGGGCTACTGGTGGT<br>GCGAACACGTCCGCAGCTACGGATGGTTCCACGCCGCTGTCTATCTCC<br>ACGAGCTCGCTGCCCGCAGGTAAGCAGAACGCGGAGTACAACGCGAC<br>CGTAGCGGTCAAGGGCGGTAAGTCTCCGTACACCTACGCGGTGTCCA<br>CCGGCACTCTGCCAGAGGGCCTGTCCTTGAACGAATCCATCGGCGCTA<br>TCACCGGTACTCCGACCGCTGCCGGAAAGTCCACCTTCACCGTGAAG<br>GTCACCGATGAGGGCAAGCTGTCGGCAACGAAGCAGCTGGCCATCAA<br>CGTCGCTGTCGCCTAA	MADQKTLEGFNARKAR<br>VGMTGAVRSSALGTEP<br>VPFGEKYNTDTHYNLG<br>YISPDGLEISFDEDKQEY<br>IPWQEVSAIRTDITKAV<br>KSVKLTLWETGIENFAK<br>FLGVSEDALEDQGDGS<br>FAFYEDALPQFGHEHL<br>HIDVVDGDKALRLDLL<br>DAQITERGSMVFKKDE<br>MFGLEVTYTSYPASYE<br>DYNATLPEGVGKTARW<br>QMNSAWATGGANTSA<br>ATDGSTPLSISTSSLPAG<br>KQNAEYNATVAVKGG<br>KSPYTYAVSTGTLPEGL<br>SLNESIGAITGTPTAAG<br>KSTFTVKVTDEGKLSAT<br>KQLAINVAVA
CSP1_44	+	23016	23378	*orf44*	hypothetical protein	ATGTCCAATATCAATCTTGACGCCCTCATTGAGCAGCGCGCAGAAGCC<br>ACCGGCTCCAACAAGGGCCGAATCCCGTTCGACTTTAAGGGCGAAAC<br>CTACGACTTTCAGGACCCGCTGACTCTGACCGACGATGACAAGGAAG<br>AGCTGCAGGCCATTGATTGGGAGCCTGACCTTGCCGCCTGGTACATGG<br>GCGATGAGCAGTACGACAAGTTCCTTGCCGCAGGCGGCTCCTCGAAC<br>CTGTGGTTCCTCGTATTCAACGAGTACCTGGAGCGTAATCAGGCCGTC<br>GATTCTTCGGGAAAAGGTACACGCCTGAATCGCTCCTCGCGTCGTTCG<br>GCGGCCCGGAAGCAGCAGAAGCGTCGCTAG	MSNINLDALIEQRAEAT<br>GSNKGRIPFDFKGETYD<br>FQDPLTLTDDDKEELQ<br>AIDWEPDLAAWYMGD<br>EQYDKFLAAGGSSNLW<br>FLVFNEYLERNQAVDSS<br>GKGTRLNRSSRRSAAR<br>KQQKRR
CSP1_45	+	23732	28510	*orf45* Smc3	Chromosome partition protein Smc/ Tail length tape-measure protein	ATGTCCGATGCTGTGTGGGTACCAGTCAATGCCGAGATGAAGGGGTT<br>CATAGGCACGCTGGTTAAGGAGGCGTCAGGCGCGGCGAAAAAGGGC<br>GGCGAAATTGTTGAGAAGGAGTTCGCCGCCAGTGGTAAGCGCGCTGG<br>TGAGTCCATGGCTAGTGGGCTGCAGGCGTCTGCGGCGAAAGTCTCCC<br>AAGTCTCCACTAAGCTCGCGACGGCGCGTAAGGCTGAGGCCCAGGCG<br>GCGGCAGACGTGGGAACTGCGGAAGCGAAGCTGGAGAATCTGCGTA<br>ACTCCGGAAATGCTAAGGCTTCGCAGTTGGCGCAGGCGGAGCAGCAG<br>CTCGAGACGGCGAAGAACAAGCAGGCCGATGCGGCAGTGCGCGTGG<br>CTCGTGAGGAGAAGAACCTCGAGGCTGCGCGTAGTGGTGGGGAGACG<br>AAGTCGCTTGCGCTGTCTCGGGCCGAGGACCAGCTGGCGAATGCCCG<br>GACGTCGCAGCAGACCGCGTCGGCGAAGATCCGCACCGCGGAGATTC<br>AGCTGGATGAGGCTCGGGCTAATGCGAAGGCTAAGTCTGATGCGCTC<br>AGGGATGCCGAGGGCAACCTCATAGGTACTCGTCGACTCTATGGCGA<br>GAACTCTAAGCAGGCAGCGGCGGCGGAGAAGGAATTCAACGCGGCG<br>CAAAAGCAGTCCGCTACGGCTAATAATCAGGTTGCTACTGCTGAGGG<br>GAAGGTGAAGCGCGCTCGTGCAGAATCGCAGAGCGCTGCAGATACCT<br>TGAAGGCCAAGGAGCTGGGGCTGAAGGCCGTGCAGGAGGATTTGGCG<br>CGGTCTTCTGAAGATGCTGCGGCTAAGACCGACAAGATGGGGGATTC<br>TTTCAAGGGGGCGGCTGGTAAGGCCGCGGACTTTGCTGGTAAGTACA<br>AGGTTCATGCTGCTGCAGCTTTGGGTGGTATTGGGCTTATCGCGAAGG<br>AATCAATTGATTACGCTTCGGAGGCCGAGCAGTCGTATGGTGCCGTTG	MSDAVWVPVNAEMKG<br>FIGTLVKEASGAAKKG<br>GEIVEKEFAASGKRAGE<br>SMASGLQASAAKVSQV<br>STKLATARKAEAQAAA<br>DVGTAEAKLENLRNSG<br>NAKASQLAQAEQQLET<br>AKNKQADAAVRVARE<br>EKNLEAARSGGETKSL<br>ALSRAEDQLANARTSQ<br>QTASAKIRTAEIQLDEA<br>RANAKAKSDALRDAEG<br>NLIGTRRLYGENSKQA<br>AAAEKEFNAAQKQSAT<br>ANNQVATAEGKVKRA<br>RAESQSAADTLKAKEL<br>GLKAVQEDLARSSEDA<br>AAKTDKMGDSFKGAA<br>GKAADFAGKYKVHAA<br>AALGGIGLIAKESIDYA<br>SEAEQSYGAVESIFGDH

						AGTCCATCTTTGGGGACCATGCTCAGGGAATTATTTCCGCGTCTAAGG<br>GCGCGGCTGAGGCGGTCGGCCTGTCCGGGCGCGAGTATCGCGAATTG<br>ACGTCGTCGACGGGCGCGATGCTGAAAAACATGGGCATGCCCATGGA<br>TGAGGTGGCGGGCAAGTCGCAGAACCTTGTGGGTGTGGCTGCCGACT<br>TGGCGGCTACTTTCGGCGGGTCGACGAAGGATGCCATTGAATCCGTC<br>AACGCGCTTATGCGTGGTGAGGCTGACCCCATCGAGAAATATGGTGT<br>GTCCATTAAGCAGTCTGACATCAATGCCCGTATGGCTGCTAAGGGCCT<br>GGATAAGCTGACTGGTGAGGCAGCGAAGCAGGCGCAGGCGCAAACG<br>CTGCTGGAGATGCTGACGGAGCAAACGTCGTCGGCTCAGGGCCAGTT<br>TGCTCGCGAGACTGACACGGCTGCGCATAAGCAGCAGGTCGCGACTG<br>CAAAGCTGAATGATGCGAAGGAAGCTATCGGTACTGGCTTGCTGCCG<br>ATGTTCGCGTTGGCGGCTGAGAAGGCGGCACAGTTCGCCGGGGTGGT<br>CGGTAAGCACCCGAAGGTGTTTATTGCTTTGGGTGCGGCTATCGGCAT<br>CGTTGCGGGTGCGATTGTGACATTGGCAGCGGTCGCCCCGATTTTTAC<br>CGCGATTAGTGGTGCCGCTGCCGCTGCGAAGATGTCCATGTGGGGAT<br>ATGTCGCTGCGCAGGCTGCAGCGATCGCCCCAATTCTTGCAGTGGTCG<br>CCGCGGTGGCTGCGGTTGGTGTGGCACTGTGGGCGTTTTTCACGAAGA<br>CGGAGACGGGCCGCAAGATGTGGTCTGCCTTTACCGATGCGCTGGCT<br>GCGGGCTGGGATTGGGTAGTCGAAAAGTTCAAGGCTGGCCTTGATTG<br>GGTTCAGTCCACGTTCGGCCCGGTGTTTTCCCAGATTAGTGAGACTGT<br>TTCCAGCGCGTGGGATGCCACGGTAGAGAAGGTCACCGGGGCGGTTG<br>ACCGCGTGAAGGAGATTGTTTCCGGGGCTCTGAACTTTTGGAAGTCTG<br>GTGACACGACTGACTACGCCGAAGCTCTGGGTATGAACCCAGATTCC<br>CCAATCTTCACGTTCCTGCAGCTCTTCCGCGACAAGCTCATTGTGCTG<br>AAGGACTTCGCCATTGTGGCGTGGGACTTTATGAAGGCCAAGTGGGG<br>CGAGTTCACGGTAGGCTTCGGCCAGTTCTATCAGACCTGGATCGCCCC<br>TGTCGTGTCCGTCATGGGCACGGTGTTCCAGGTGCTGGGCACCGTGGT<br>GATGGGTGCGCTACAGGGCATCTGGACGGGGATGCAGTTCGTCGGCA<br>GTGTCATTTCGACGGTGTGGTCCTCGGTGATTCAGCCAACGCTGTCAG<br>TCTTTATGTCGGTGGTGCAGGCCGTGGGCTCGTTTGTCGCCCCAATCT<br>TCATGGCCATCATTGGTGGCGCGTTCCGCACCATGGGCACGCTCATCT<br>CCAGCATCTGGAGCGGAGTCATTAAGCCCGCATGGGATTTCTTCCGCA<br>ATGCAGCCGGATTGCTCGCGGACGTGCTCACAGGCAACTTTTCGAATA<br>TCGGGAATCGTTTCTCGTCGATGGGTGACGCCATTCACAACGTGGTGC<br>GCGGCGCCATCAACACGGCGATGAACCTCTTCAAGTCCATCTTTGAGGA<br>CAGCGAAGGGCGTGGCTGCAGCATTCGCCCAGTCCATCGGCAACATG<br>GTCAACTCAGTCCGCGGCAAGATTGGCGAGATGATGGGCGTGCTCGG<br>CCAGATTCCGAGCAAAATTCAGGGCGTTTTCGCCTCTGCTGGTACCTG<br>GCTGGTCAACGCCGGCAAGAACATTATCAGCGGTCTCATCAACGGCA<br>TTAAGTCGATGTTCGGGCAGGTAGGCAACGCGATTGGTTCGATTATGC<br>CGGACAAGATTCGCGGCATGCTCGGCTTCATGGACGGCGGCGTCGTT<br>ATGGCCGAGGGTGGTATCACCCGCGCCTACATCGACGGTGGTATCGA<br>CAAGCTAGAGCGCTACGCCAACGGTGGCTCTCGTGAGGACCACCGGG<br>CGCAAATCGCCAAGGGCGGCGAGTGGCGTGTGTGGGCGGAGCCGGA<br>AACCGGCGGCGAATCCTACATCCCGCTAGCTAAGTCGAAGCGGAAGC<br>GCTCTACGCAGATTCTTGCCAAGACTGCCGATATTTTCGGCCTGTCTG<br>TCGTGGACAAGAAAGGGCAGCGTGTACAGCCCGGCTCTGCTTCGCAG<br>GTAGCCCCGGCACGAGCCCAGTATTTCGCAGACGGCGGTATCACTGC<br>TAAGACTCTGCGTGATTTCGTGGAGGGTCGTCCTGCTAATGCTGCTGG<br>AATTAGCCCGTTCCCTCGTAAGTTCGCTACTGGCAATGAAGCCGCGTG<br>GCTGTCCTCGCACGGCTTCCGTCATGGTCGCGGTAAGCAGGGCGATCT<br>GCGTTTTGGCCTCAAGAACGGTGGCCCTGCGGGCGGGCACACCGCCG<br>GTACCCTCCCGGACGGTACGAACATCGAGATGGGCGGCGGTCGCGGT<br>AACGGCCAGATTGGTGGCCGTGCCGCTGGAGCATGGGACTCCTATTTT<br>AACGAGTTCTTCTACAAGACCATTCAGCCGCCGAAGCCGCCGAAGAT<br>GAACACCATCCTCTCCCAGAATGCGCTACCGGACGGCACGAGCATGA<br>GCATTGACGGCGTTCCCGTTTCTGTGAGTGCAGATGAGGCCACCGGTA<br>CAGGGGAGAGCACGGTTACTGTCGCATTGTCTCCGGAGGATGCGGCA<br>CAAGCTGCGTTCTACAAAGAGCTCGGAGATAAATCACTGCTTGACAT<br>CGCAGTCGATGGTGTCTTCGATTTCTTTGGCATGGGTAGCTCACTGAC<br>TAAGAAGCTTCTGACCACGAAGGGAAGCGAGTTGGTGCCTCAGCAGG<br>CTAGTGCGACGTCATCATCGACGGTGTCTGCTGCAACTAGTGGCCGGG<br>ACCAGGCGCGCACTAGCGTGGCGGCGAAGAACGCCAAGGCGATTGA<br>GGATGATGCGCTTAAGAACGCGTCGCCGTCGACGATTGCTAAGGATG<br>CTCAGCTGCGGATTGAACCGGATAAGAAGGCCAAGACTGCGGAGTGG<br>GGCCAGGAGTTCTTCGTGAGTGAGATTGCGGAGGCGGCGAAGCGTCT<br>TGGTGTGGGTGCTAAGGGCGCGAAGATTGGTGTGGCTACGGCGCTTG<br>TGGAGTCGGGTAATCCGATGAAGATGTGGGCCAACAACGCAGTGCCG<br>GAATCGCTCAAGTACAAGCATGACGCGGTCGGCTCCGACCATGACAG<br>TGTTGGCCTGTTCCAGCAGCGTGACAATGGTGCGTGGGGCGAGGTAT<br>GGCAGCGCATGGCCCCCTACGAGTCTGCTGCTATGTTCTTCCGCCAGC<br>TGCATACCTTCAAGTGGCAGGACATGGAGCCTGGCGCTGCGGCGCAG<br>AAGGTTCAGGTGTCCGCATTCCCGGGCCGGTATGCGCAGCAGATGGG<br>ACTTGCCGAGGAACTGGTCAATAAGACCGGTGTCTTCGACAGCGGTG<br>GCCTCGGCTACGGCAAGGGCCTACTACCGAAGGATGTAGTCGCTCCG<br>GAGCGTGTTCTGTCTCCGCAGCAGACTCGCGCTTTCGACGACCTTGTG<br>TACAAACAGCTACCTAAGCAAAACGGCGAAGGCGGCAGAGGTGACA<br>CGACGATTGTCGTCAATTTGGATGGGGAAGAGATTGTGCGTGAACGG<br>CTTGAAAAGGCGGAAGGAAAAATTGAGTTCAATGCGAACGAGCTCGC<br>AAAACTCAAGTTCAAGCCACAAGCTGTTACAGCTGCAATCAACATGA<br>TGGTTTAG	AQGIISASKGAAEAVGL<br>SGREYRELTSSTGAML<br>KNMGMPMDEVAGKSQ<br>NLVGVAADLAATFGGS<br>TKDAIESVNALMRGEA<br>DPIEKYGVSIKQSDINA<br>RMAAKGLDKLTGEAA<br>KQAQAQTLLEMLTEQT<br>SSAQGQFARETDTAAH<br>KQQVATAKLNDAKEAI<br>GTGLLPMFALAAEKAA<br>QFAGVVGKHPKVFIAL<br>GAAIGIVAGAIVTLAAV<br>APIFTAISGAAAAAKMS<br>MWGYVAAQAAAIAPIL<br>AVVAAVAAVGVALWA<br>FFTKTETGRKMWSAFT<br>DALAAGWDWVVEKFK<br>AGLDWVQSTFGPVFSQI<br>SETVSSAWDATVEKVT<br>GAVDRVKEIVSGALNF<br>WKSGDTTDYAEALGM<br>NPDSPIFTFLQLFRDKLI<br>VLKDFAIVAWDFMKAK<br>WGEFTVGFGQFYQTWI<br>APVVSVMGTVFQVLGT<br>VVMGALQGIWTGMQF<br>VGSVISTVWSSVIQPTLS<br>VFMSVVQAVGSFVAPIF<br>MAIIGGAFRTMGTLISSI<br>WSGVIKPAWDFFRNAA<br>GLLADVLTGNFSNIGNR<br>FSSMGDAIHNVVRGAIN<br>TAMNLFKSIFEAAKGV<br>AAFAQSIGNMVNSVR<br>GKIGEMMGVLGQIPSKI<br>QGVFASAGTWLVNAG<br>KNIISGLINGIKSMFGQV<br>GNAIGSIMPDKIRGMLG<br>FMDGGVVMAEGGITRA<br>YIDGGIDKLERYANGGS<br>REDHRAQIAKGGEWRV<br>WAEPETGGESYIPLAKS<br>KRKRSTQILAKTADIFG<br>LSVVDKKGQRVQPGSA<br>SQVAPARAQYFADGGI<br>TAKTLRDFVEGRPANA<br>AGISPFPRKFATGNEAA<br>WLSSHGFRHGRGKQGD<br>LRFGLKNGGPAGGHTA<br>GTLPDGTNIEMGGGRG<br>NGQIGGRAAGAWDSYF<br>NEFFYKTIQPPKPPKMN<br>TILSQNALPDGTSMSID<br>GVPVSVSADEATGTGE<br>STVTVALSPEDAAQAA<br>FYKELGDKSLLDIAVDG<br>VFDFFGMGSSLTKKLLT<br>TKGSELVPQQASATSSS<br>TVSAATSGRDQARTSV<br>AAKNAKAIEDDALKNA<br>SPSTIAKDAQLRIEPDK<br>KAKTAEWGQEFFVSEI<br>AEAAKRLGVGAKGAKI<br>GVATALVESGNPMKM<br>WANNAVPESLKYKHD<br>AVGSDHDSVGLFQQRD<br>NGAWGEVWQRMAPYE<br>SAAMFFRQLHTFKWQD<br>MEPGAAAQKVQVSAFP<br>GRYAQQMGLAEELVN<br>KTGVFDSGGLGYGKGL<br>LPKDVVAPERVLSPQQT<br>RAFDDLVYKQLPKQNG<br>EGGRGDTTIVVNLDGE<br>EIVRERLEKAEGKIEFN<br>ANELAKLKFKPQAVTA<br>AINMMV
CSP1_46	+	28520	29305	*orf46*	hypothetical protein	ATGGCTAGACCTCGAGTGAAGTGGTTCGGCGTCGATGATTCAACGTG<br>GGTGCTGGCGAGGGACGGCATGACTTCAAATGGGGTCATGCTAACCG<br>CCGTCAGTGGTCTTGTTGCGAGCCCTTCGCGGAAGATTACGCCATCTA<br>CTAATGGGGTAGGCGTGGAGTTTGGCAGGTCGACGTGGCCGATGCTC<br>GAGGGAGAGCTAAAATGTCGAGTCTACGCCCGCAAAGACGAAACTGT<br>GCAGGATACTTATTCACATTTTCTTGCGTCCTTTTCGACATTTTCCCCT<br>GGCAGATTGTCTATTTCGATGACGGGCCAGGCGGAGTGGGAAGCCGA<br>GTGCCTCCTTAAGGAGAGTATCGGAGCTCCCGAGAAATCTCCTGCGG<br>CGTCGGGGCTCATTTCCCTCGACATTGAAATACCCCTCATCTGTAATG<br>CTGGAGCGTTTTCCTCTCCGGTGGAGCGGATTTTCGATTCGAAAGCCA<br>TTGTGCGCAACATTGGTGACCTTCCGTTGTTCCCCAATGTGGTGTGGT<br>CCGGTGCAGGGCAAACTGTACGGCTGCCCAATGGTGTCCTGGTGACT<br>CTGCCTACGGTGAGTGGTGAGCGTGTTTTGTCTACTGACCCTGGTCGT<br>GGCTATGTGGTGACTGACCGTGCAGGTAAGCCGGATAAGGCTGCGTG<br>GGCGTCGCTGCGGGGGTTAGCGGTACCTGGTGAAACGTTGCCGGGTA<br>ATGCGACTTCGTGGGAGTTATCCAGTGGTGTGCATCTTGAAGTGGTGC<br>AGCGTCGAGAGAATCCGTGGAGGTAA	MARPRVKWFGVDDST<br>WVLARDGMTSNGVML<br>TAVSGLVASPSRKITPST<br>NGVGVEFGRSTWPMLE<br>GELKCRVYARKDETVQ<br>DTYSHFLASFSTFSPGR<br>LSISMTGQAEWEAECLL<br>KESIGAPEKSPAASGLIS<br>LDIEIPLICNAGAFSSPV<br>ERIFDSKAIVRNIGDLPL<br>FPNVVWSGAGQTVRLP<br>NGVLVTLPTVSGERVLS<br>TDPGRGYVVTDRAGKP<br>DKAAWASLRGLAVPGE<br>TLPGNATSWELSSGVH<br>LEVVQRRENPWR
CSP1_47	+	29309	30922	*orf47*	putative tail protein	GTGTCTGACTGGGCTGATGTCCGTGATGCTGCCCACCGTACTGGTGAT<br>GGCTGGGGGTGGTGGCTGCTGGATAAGAACATGGAACCGCTCGCTGA<br>TCTTCATGGCTGCGTGTCAATTGAAATTCCGGAGTCAGTGAACGAAAC<br>GTCTGTGCTTAAGCTTGAGCTGCGGGATGATCATCCGGCCTTTGATTT<br>TCTTTTGCCGTTTGACATGTTGGACCCAGGCGCTCCGGAGCTGACGTG	MSDWADVRDAAHRTG<br>DGWGWWLLDKNMEPL<br>ADLHGCVSIEIPESVNET<br>SVLKLELRDDHPAFDFL<br>LPFDMLDPGAPELTWR

						GCGAGCATTGGTGCATGAGTCGCAGTGGATCATGGTCGAGGGCCCACGCGGTGCGGTAGACCGTCTGGTCTATCGGGTGGCTCGAATCACTGACTCCGTCGGCAATGGAGAAGCACACGGAACCGTCACTGTCGAAGCTAAGTCTTTGTTCCGATACATCGAAAAAATTGCTCTTCGTGCAAGCCCCAATGACCCGCTTATCGCACAATTGAAGTATCGAGACTTCCGCGCCGGAGACTCCCTTCGCGTACTAAAAGAGTATCTGCTCGTCAATCTGATGCGGGACTTCCAACCACGTGCCATCAAAGGGTGGAATCTGTGGTCCTCTAACGCCTGGGCCTCAGTACGCCCTGACGCCTGGCCTGCCATCGTGTCGCCAGTACATGAATCCACATCGACGCAGGTGACAGTTCTTGACGCCCGCTTCGACATGGCGGGAGATTTCTTCAAAGATACACTCGATGCGGCTGGCCTCATGCTGTCTATTAACCTATGGCTGCCTGGGGACGAGCAGCCGGCGCCTTCCCACGTACAACTACGGTTACCTACTTTGTGGATAGACATTGTGCCGCGACAGTATGATACGTCAACAACTGGCCATGCCCTTGATCTATTCCGAGGGCTGATACGCCAATTTGACCGGGAAGCCAACGCTCCGCGTATTGGCATGGGGACGACACCAGCAACTCTGGATCATCTTTTACCATGGGTGGTGTGGAGACCTGAAGACATGGCAGGCATTACTTCCGATTTTACCGTCGTGATAGTCGGAGGACTGGCACGTTACCGTGGGTGGTCGATCCCCAGAGATCGTCAACAAGCTCATCGGGGCTGGCTCGAAGGCGTTGTTTAGCGGCTTGGCTGCTGGACTAGCTCAATTAGTTCCGGTGTTCGGGCCGCTCATCGCCGCTGCCGGTGCGTTCCTTGGCGAAATATCCGCGGCAGCCCTGAAGGACAAGCTTTTCGCATGGAATGAGTTTTCCGACACCGTCAGGCGTGCGGCGCATGGACGATTCGCTTACCGAGACCAGGTAGGAGCAGGTGATGGCTGGTCGCTAAGTGCATTCCAGCAGGGCTTTGAAATGTTGCAGCAGGGCGCGGGCATGGTCAGCATTGGCTTCCAAGTCTCGGAGCAAACCGTCTACCGGTGGGGTGAGCACTACCGCGCCGGGGACCAACAAGGGGTGGTCCACCGAAGCGTTGTTTTTGCTACGTACGTCAGTAACGCGACGCTGCATTGGTCCGTAGCAGATGGATGGAAAGAAGAGCTGACGTTGGGTGATCCGCGGGCTCGTGAGTCTTACGAGCGTGGGTACACACGGTCGTTGAAATCCATTTCAAATGCCATTGATCGTGTGAAAACTTTCGTTCACTAG	ALVHESQWIMVEGPRGAVDRLVYRVARITDSVGNGEAHGTVTVEAKSLFRYIEKIALRASPNDPLIAQLKYRDFRAGDSLRVLKEYLLVNLMRDFQPRAIKGWNLWSSNAWASVRPDAWPAIVSPVHESTSTQVTVLDARFDMAGDFFKDTLDAAGLMLSINLWLPGDEQPAPSHVQLRLPTLWIDIVPRQYDTSTTGHALDLFRGLIRQFDREANAPRIGMGTTPATLDHLLPWVVVWRPEDMAGITSDFTVVKSEDWHVTVGGRSPEIVNKLIGAGSKALFSGLAAGLAQLVPVFGPLIAAAGAFLGEISAAALKDKLFAWNEFSDTVRRAAHGRFAYRDQVGAGDGWSLSAFQQGFEMLQQGAGMVSIGFQVSEQTVYRWGEHYRAGDQQGVVHRSVVFATYVSNATLHWSVADGWKEELTLGDPRARESYERGYTRSLKSISNAIDRVKTFVH
CSP1_48	+	30935	31321	*orf48*	hypothetical protein	ATGCACATAGATGATATTTATCCGTACGATAAGGACCCTGACCGGCATCCGCTTTCTTGGGTGTTTTTTAATGCAGGATTCGCGTTGACTCTTGATGCTGCGGAGAAGATGGCGAAGCATGTTTTCGAAGATTTGGGCTGCGGCGGACCTGGTAGTGCACATCCGCCTGTGGTGAAGTATGACGCGGTGGGGTCGTCGGGCGCTCCGTGGGAGCCGGGAGTGTGGATTTCTTTTGATGAGCCGCGGATGAGTTTTGTCGCGACAGCTCCGGATAAGGATATTACGGCGATGAGTGCTGAGGAGCGCGCAGAGTTGCGTGAAGCTTTGGCGGTTGCGGAGAGCATGGACGCCGCCAAGCGTCACAGTGACGTAAACGGGGAGGGCTAG	MHIDDIYPYDKDPDRHPLSWVFFNAGFALTLDAAEKMAKHVFEDLGCGGPGSAHPPVVKYDAVGSSGAPWEPGVWISFDEPRMSFVATAPDKDITAMSAEERAELREALAVAESMDAAKRHSDVNGEG
CSP1_49	+	31325	32062	*orf49*	hypothetical protein	ATGGGTGTTCATCTTGGCCCCTCGCTTCCGACGGATCCGAACTCCCGATCTGCTCTAGGCGGCATCGATGCTTTCGGTGGAGGATTGGGCGACGCTCTCGGTAAGGGCTTCACTGGGCTCATACAAGGTATTGGTGATGCGCTCCGCGGTATTTTTGAGCCTGGGGGAATCTTCTCCCCAGTGGGTGAAGCTGCCCAGCAGATACGCGATGGTCAGCTGGACTTAAATGACCGGGTTGATCTTCTTTCTCCGATTCAGGATTACGGGTCCGTTTTTATGCCTGCGGGCCGTGAGATAAAGGGGCCTAGGACGCTACCGTTTACGGAGCAGCTGGGGCCGATGCGCGGGTGCTCAAAATCATCTATGGGAATTCGGCTTGATGATATTGGCCTGTGGGATATTCGGGCGCAAATCACAGTCTCATGGGTGCGAATTGTCACCGGTGACGTGAACTGGCGAGTAATCGTCTACCGGCCCAACGGGACTATTTATTCACAGCAACTTTCGTCATTATCGGGCAACGGAAATATGACTGGAACGATCGTTTCCTCTGTGGTGATTGATACTCCTGGCTGCTATGTCGAGGTCAAAATGGATTACATCGGTACGGGTCGCGAAATTTTGGGAGGACCTGGATGGACTCGCCTCGTGGCTCAACATATTTCACGCAAAGTCGACGGTGAATGGGCTCGAGGTACAGAAAAATCAGATAATGCAAGCCGGCCTGGGGAAGGATAA	MGVHLGPSLPTDPNSRSALGGIDAFGGGLGDALGKGFTGLIQGIGDALRGIFEPGGIFSPVGEAAQQIRDGQLDLNDRVDLLSPIQDYGSVFMPAGREIKGPRTLPFTEQLGPMRGCSKSSMGIRLDDIGLWDIRAQITVSWVRIVTGDVNWRVIVYRPNGTIYSQQLSSLSGNGNMTGTIVSSVVIDTPGCYVEVKMDYIGTGREILGGPGWTRLVAQHISRKVDGEWARGTEKSDNASRPGEG
CSP1_50	+	32066	33331	*orf50*	hypothetical protein	ATGGCGGTAACACTTACAATTGATATAGGAGACTTTGGTGCTGAGTCGCATCCTGATGATTACGTCATCTTGTACGCGCCGGCGTTCCGAGAATCTGCAGAACGCTCTGGGGGTCTGGTGTCTACGGCGCCGCGGAGGGTTTACCTTACTGGGGGGAAAGCGGCGGTCGAAGTTGAGCCAGGGCCGCTTGCGGTGGAGTTCTGTGTGCGCAATATTAAAGATTCCTCGACTCGAGAGTTTGTGGTCCCTGCTGGTGGAGGAAGTCTTGGCTCCTTGCTTGCGGCTTCGCTGGATTATGAGCCGGTGGTAGTTACACGTCTGCAGGAGCTGATCGATTCGGCCGGGGATGCTGCGGAACGGCTTTCTGGTGCGGCTTTGTCGTCGGCGGAAAAGGCTGATTCGTCCGCTAAAGCGGCGAGGAGGTTTGAGGATGCGGCTTCTAAGTATGCGGCTGCGGCTAAGGTGTCTCAGGATGCAGCAAAGGGGTCTGAGGATGTGGCGGCGCAGTCGGCTTCTGCGGCTGATGTGTCTGCTAAGGCAGCTAAAGCTTCGGAGGATGCGGCGGCAGGCTCCGCTTCATCTGCTAAGCGGTTGGAGTCTAACGCGGCGGCGTTTAAGGACGCGGCCGACAGCTCCGCGTCCGATGCCGCTACCTCTGCTGGTAATGCGAAAAAGTCTGAGGATGCGGCGAAAGCGGCACAGGCACGTTCCGAGGAAATCGCTTCTAGTACCTCGTGGGATGGTGACAAGCTGACCGTGAATGGTAAGACGTCTCCGTCGCTTACTGGCCCGCCGGGCCCTAAAGGCGAGACCGGCAGTGTAGAAAATGTTTCGTGGGCTGATATTTCGGGTAAGCCGGATTTTGCTTCTACCTGGGAGCAGGTGAAGGGCAAGCCGGATGCGTTCCCTACTACGTGGGATGAGGTGAAAGACAAGCCTGAATCTTTCCCACCAGGAAAGCACGAGCACGAGATTCGCGAGGTAAACGGCCTTACTGAGCTCTTGCTAGATAAATCCCCCCGTAGTCATAGGCATACCCTGTCGCAGATTAGTGACGCCCCCAACACGCACACGCCTTCCGCCGTTCCCGACACGCTGATGTCGCGTGATGCTTACGGTAGGGCGCATATTTCGACCCCAGTTCACGACGGGCAGATCGCCAATAAGCGGTATGTGGATGCTGCGGTGTCTTCGGGTAGCAAAATTAAGGTTGTGTCGTCGCTGCCGTCGTATCCGGATTCTTCGACTGTTTACATTGTGGTGTAG	MAVTLTIDIGDFGAESHPDDYVILYAPAFRESAERSGGLVSTAPRRVYLTGGKAAVEVEPGPLAVEFCVRNIKDSSTREFVVPAGGGSLGSLLAASLDYEPVVVTRLQELIDSAGDAAERLSGAALSSAEKADSSAKAARRFEDAASKYAAAAKVSQDAAKGSEDVAAQSASAADVSAKAAKASEDAAAGSASSAKRLESNAAAFKDAADSSASDAATSAGNAKKSEDAAKAAQARSEEIASSTSWDGDKLTVNGKTSPSLTGPPGPKGETGSVENVSWADISGKPDFASTWEQVKGKPDAFPTTWDEVKDKPESFPPGKHEHEIREVNGLTELLLDKSPRSHRHTLSQISDAPNTHTPSAVPDTLMSRDAYGRAHISTPVHDGQIANKRYVDAAVSSGSKIKVVSSLPSYPDSSTVYIVV
CSP1_51	+	33583	33834	*orf51*	hypothetical protein	ATGCTCTATGTAGGCAGCAAATACCCCTCAAATGTGGCAGTGGGTAGCAGGAGTGCGAAAGCGGTATTCCGTGGCAGCACCCCACAATGGGCACCCCCATGGCAGGCCACCGCAACCTACGCCGTGGGGGATATCGTCGCGAGCCGTGGCGGCTTCTGGGAGTGCATTAAGACGCACAAATCGAAGAATATTTACGAGCCGGGCAGTGACTACGGATCGCAGGATTACTACTGGCGCATGTATCGACAGTGA	MLYVGSKYPSNVAVGSRSAKAVFRGSTPQWAPPWQATATYAVGDIVASRGGFWECIKTHKSKNIYEPGSDYGSQDYYWRMYRQ
CSP1_52	+	33867	34439	*orf52*	hypothetical protein	ATGACTATTGAGCAAATCCAAGCGCAAATTCGGGCACTCCCGGATGATGAGTACATCGACCTGCGGGTATGGATGGGTAGTGAGGAACGCGCCCGCCGTGAAGCACAACCCGCCATTGAGCAGGCACAGGCCGACCTGGTGAGCGAGCTACAAGACGCAGGCAAGCTGGAGAAACCGGAGGCGGTGACGCTTGAGGAAGCTATCGCGGCGCCGGACAAGGTGCCAGCGTGGGAGAACCCGCTCACGGATCACTCGAAGATGTACGCGAAGACCGATGTCATCACCCACAACGGACACTTCTGGGAGTCCACCCATGCGGGCCTTAATTCCTGGGAGCCTGGAGCGCAGGGAGTGGACGAGTACATTTGGCGAGATGTCACCGAGCGGGTGCGCCCCACCGCACCGGAGGAGAACACGGCTAGCCCTGGCGCTATCCCGTTCGCCCCCGGGCTACCAGTGAAGGAGGGCGACCTCGTGGAGTATGAGGGCGTGGTGTACAAGGTGCTCAGCACCCACACCACACAGTCCTACTGGCCGCCCAGCGAGTCGCCAAGCCTGTTTGAGCGCGTGTAG	MTIEQIQAQIRALPDDEYIDLRVWMGSEERARREAQPAIEQAQADLVSELQDAGKLEKPEAVTLEEAIAAPDKVPAWENPLTDHSKMYAKTDVITHNGHFWESTHAGLNSWEPGAQGVDEYIWRDVTERVRPTAPEENTASPGAIPFAPGLPVKEGDLVEYEGVVYKVLSTHTTQSYWPPSESPSLFERV

CSP1_53	+	34509	35879	*orf53*	lysozyme M1 (1,4-beta-N-acetylmuramidase)	ATGGTTACTATGCCGGTTGATAAGGGCTTTGTGGTCACTAGCCCGATG<br>GGGCCGAGATGGGGCCTATATCACTGGGGCGTGGATTACGGCGTGGC<br>TGGCGGCTCAGGCGGCAAGCCAATCTACGCGATTAAGGACGGCACCG<br>TTATTCAGGCCGGGGCAGCGTCTGGTTTCGGCCAGTGGATTCGTATTG<br>ACCACCCCGCCAGCGTTGGGGGTAATGAGTCAGTCTACGGGCATATT<br>ATCCCCGAAGTTCGTGAGGGGCAGCAGGTGCGTGAGGGGCAGCGTAT<br>CGGCAGGATTAATCCTAACTCGGCAACTAACGGCGGTGTGGCCCCTC<br>ACCTACACATTGAGGTGTATAAGTACTCGTGGGTTGGCCCGGGCCAG<br>CGAGTAGTGGGGCAAACGATTCTCGACCCGCAGCAGGTTCTACGTGG<br>TGCGAAGTGGCCCGGCGAGTCCACCGCTCGCCCTGTGGGGAAGCGGG<br>ACGGCACTATTTTCGGCGTGGATGTATCTTCTCACCAGGACGGAATGA<br>GCCTGAAACGCGCCGCCAGTGAGGGGATTGACTTCGCGATTATCCGC<br>ACCACGGACGGCACCTATAAGGATCGCTGCTATCGTTCTCACCTTGAT<br>GACGCGGAGAGCGCGGGCCTAATCACGGCGGCGTATCACTATCTCCG<br>CAACCCTAGCGAGGGCACCACCATAGCGCAGCAGGTACAGGCAGCAC<br>TTGAAGTCATGGGCGACAAGAAACGCCCCATGTGGCTTGATTGCGAG<br>ACGCCCGCCGGTTTGCACGTTGACCACATCCGCGAAGCGAAGCGCGA<br>GTTTGAGCGCCGCGGCGTGCGCGTTATCGGCGCGTATTCCTACGTGCC<br>GTATTGGGAGGGCAGCATCGCCCCCGGCGAGCCGGACAGCCACGAGT<br>TCGGCGCTTTCTGGGTCGCGGCCCACGGGTCAAACCGCACAGGCACG<br>CCAGCCAATATTTACCCCGGCGACAGCGCTAGCCAGTGGGACTACCC<br>GCTCGGCAATCAAAAACCCGCACTGTGGCAATACGGCAGCAATGCGC<br>AGGTTGCGGGCTACAACGTGGACATCAACGCCTACCGCGGCAGCCGG<br>GATGAGCTACGCGCCCTGTTCTACGGAGGTAAACAACATCATGAGGG<br>AGAAGAAATGACCACTAAATTTTTCACCGACTTTCTGACCGGCTATTT<br>AGGTCCTCAGATTAAGGCCATTCAGGAAATCTGGACTCAGCTACGCG<br>GCCCCGGCGGCAAAGGCTGGGAGCAGCTAGGCCAAAACGCGCAGGG<br>ACAGAACCTCACCCCGGTGGACGCGCTCGCGGCTATCCGCCAGCAGC<br>TCGCACAAATCCAGGCAGACGTGAACGGAATCAAGAGAGGCAAGAA<br>GTAA	MVTMPVDKGFVVTSP<br>MGPRWGLYHWGVDYG<br>VAGGSGGKPIYAIKDGT<br>VIQAGAASGFGQWIRID<br>HPASVGGNESVYGHIIP<br>EVREGQQVREGQRIGRI<br>NPNSATNGGVAPHLHIE<br>VYKYSWVGPGQRVVG<br>QTILDPQQVLRGAKWP<br>GESTARPVGKRDGTIFG<br>VDVSSHQDGMSLKRAA<br>SEGIDFAIIRTTDGTYKD<br>RCYRSHLDDAESAGLIT<br>AAYHYLRNPSEGTTIAQ<br>QVQAALEVMGDKKRP<br>MWLDCETPAGLHVDHI<br>REAKREFERRGVRVIGA<br>YSYVPYWEGSIAPGEPD<br>SHEFGAFWVAAHGSNR<br>TGTPANIYPGDSASQW<br>DYPLGNQKPALWQYGS<br>NAQVAGYNVDINAYRG<br>SRDELRALFYGGKQHH<br>EGEEMTTKFFTDFLTGY<br>LGPQIKAIQEIWTQLRG<br>PGGKGWEQLGQNAQG<br>QNLTPVDALAAIRQQL<br>AQIQADVNGIKRGKK
CSP1_54	+	35879	36250	*orf54*	putative membrane protein	ATGGCACGTCACTACAATCAAGCCCTGATTAACGACGTTTCGGGGCG<br>CATCGCAGTAGCGGTAGCAGCGGAGCTTAGGGAGCAGCCGTGGTGGC<br>TACGCTACAAAGGCTCAATCATGCTTGCTTTGCAGGCCCTAGCGTGGG<br>TCGCTGGCGCGCTGCCCGTCATGCTTGCAGATGCCCCGGAATGGACTA<br>TTTTTGCGGCTGGCGCAATCGGTTATTTCATCACCGCGCTAGTCAACC<br>GGCTCACCGTCGATGGTGTCACCCCAAGCATGGCGCCGCGCCTTGCA<br>ACCCAAGCAGAATACCAGGAGCAGAAAACCGCCCCGCAGCTCGCAAC<br>TCTGCCGGTCTACACCGGCCCCACCACCAACGAGGCATGA	MARHYNQALINDVSGR<br>IAVAVAAELREQPWWL<br>RYKGSIMLALQALAWV<br>AGALPVMLADAPEWTI<br>FAAGAIGYFITALVNRL<br>TVDGVTPSMAPRLATQ<br>AEYQEQKTAPQLATLP<br>VYTGPTTNEA
CSP1_55	+	36250	36672	*orf55*	putative membrane protein	ATGACTCGCACGCTTCAATGCATTATCGGGGCGCTCGCATTTCAGATG<br>CTTTCGCGAGGCATGGATTACATCCTCGGCAACCCAAACCAGGGGGT<br>GGGCGCGTTCCGTGTTCGTGATGTTACCCCGCCTGTCGTGTGGGGTGC<br>GGCCTGTATCATCGCAGCCCTCATCGTCGCTATCGGGCTACTAAAACA<br>GTGCCCACGGATTGTGCGCGCCGGGGCTGTCCTCGCCGCCGCGATCTA<br>CGGCGCTTTCGCCGTGATGGTCTTCGATGACGTGTACATGCAAGGGCC<br>AGTGGATGATTGGCGATTCTTCACCGGGTATATTTCCGCCGCGTTCAT<br>GTGGGCTGTGATCGCGTGGTCACTCACGATACGGATCGCCGTAATCA<br>AGCATAGGAAGGGGAAGGATGGAGACCACAGATCTGCTTAG	MTRTLQCIIGALAFQML<br>SRGMDYILGNPNQGVG<br>AFRVRDVTPPVVWGAA<br>CIIAALIVAIGLLKQCPRI<br>VRAGAVLAAAIYGAFA<br>VMVFDDVYMQGPVDD<br>WRFFTGYISAAFMWAV<br>IAWSLTIRIAVIKHRKG<br>KDGDHRSA
CSP1_56	+	36650	37078	*orf56*	putative membrane protein	ATGGAGACCACAGATCTGCTTAGCTTCCTCACCAACTCTAGGACGCCG<br>GGCGGCTTCCTCCTCACCATCCTCATCATCCTCGCCCTGGTCTCAGGG<br>TTGATGTCTAAGGCCGCGGCGGACTATGGCGGTATCTTTGGTAAAGCG<br>GCCCGCGCTATACGCCAGCACAAAGAGGACGCTATCGCCGCGGATGA<br>AGCCAGCGACGCCCGCCGCCTAGACCGCCTGGAGAAAACAATCCAGC<br>GCCTCGATAGGGAAGTGGCTGAGCTGCGGACGAAAGAATCCCGACAC<br>CATGAGTATCAATTGTGGGTTGCTGGTTTGTGGCGCGGCTTGGAGTTC<br>TGGGCGGTAGATAAGGGCCTGACGCTACCGCCGCCACCATTCATGAG<br>CTACCCGGAGTGGATAAAGCAAAAATACCCGGAGACGACACAGAAG<br>TAA	METTDLLSFLTNSRTPG<br>GFLLTILIILALVSGLMS<br>KAAADYGGIFGKAARA<br>IRQHKEDAIAADEASDA<br>RRLDRLEKTIQRLDREV<br>AELRTKESRHHEYQLW<br>VAGLWRGLEFWAVDK<br>GLTLPPPPFMSYPEWIK<br>QKYPETTQK
CSP1_57	-	37774	37121	*orf57*	hypothetical protein	ATGAAACCCGGCGACAAAGACGGACACGGCCGGTACGGCTACCTCGA<br>TGGTGATGATGCGCGCATCATCTGCCATGAGTGCGGGGGGCTGTACC<br>GGGCGTTGGCCCCGCACCTTATTAAAGCCCACGACATGACCGCAGCA<br>GAATACAAGCAAGCGCACGGGTTGCCACGCGGCATGGGGCTAGTAGC<br>CCCTGAGACGAGGCGCGCCAAGTCACGCCAAGCCCTAAGCCATGTGG<br>GTACACCGGAGTGGGATCGGATGGTAGAAAAGCGCGACCCCACAGCC<br>GCATCACACGCCCGAACAGAAGAGTCATTCACCACCCGTGGTGTGGT<br>TGCTGAGCAGAAAACAGCGACGGCGCGAGAAAACATCAAAGGGGTG<br>AAAAAGCCTGTCACACGCCGCTGTATAGTCTGCGGAAAGCTGCTTACT<br>GAGGTGCGCGGGCGCGCCACATGTAGCGACCGGTGCTATCGCATTCA<br>GCTGTATGAGCGGACCGCGAAGCCAGGCGCGAGGGCATGGATGCAGC<br>GCCGAGACGCGGGCGAATCACTATCGGAGATAGGACGTAGCGCGGGC<br>GTATCCCATGTGGCGGTGCGGGTGCGGATTGAGAGATTCCGGGCCTA<br>TCTCAGCTTGTGCGAAGAGCTTGGGCGCACACCTATAGAGTAA	MKPGDKDGHGRYGYL<br>DGDDARIICHECGGLYR<br>ALAPHLIKAHDMTAAE<br>YKQAHGLPRGMGLVAP<br>ETRRAKSRQALSHVGT<br>PEWDRMVEKRDPTAAS<br>HARTEESFTTRGVVAE<br>QKTATARENIKGVKKP<br>VTRRCIVCGKLLTEVRG<br>RATCSDRCYRIQLYERT<br>AKPGARAWMQRRDAG<br>ESLSEIGRSAGVSHVAV<br>RVRIERFRAYLSLCEEL<br>GRTPIE
CSP1_58	-	38175	37771	*orf58*	hypothetical protein	ATGACCACCCAGACTTTTACCCTCCGAGATATAGCCATCGCCGCCAAC<br>GCAAAGCACGGAATGGACACCACGGCCGCTGAGGACATCGCCCGCAC<br>CTACCTTGACCAGATGGACGCTGAAGACGGTATTGAGCGCGATGAGG<br>ACGAAATCACCCAAGATGATTTCGATTTCCTGCTGGGCGCTATCGATT<br>CCGCCCGCCGCGCCGGTGACCTCGGACTGCATGAGCTAGACCTGATTT<br>CCGAGGCCGCGCTTGAAATGCAATCGCAGGAGGACAGGCTACGCGCC<br>GCCCGTGATGAGCGTGACGCCGCTATCCGCGCCGCTGTCCACGCCGG<br>GGCACGCATCCAAGATGTGGCCACCGCTGCGGGTATCTCCCGCCAAG<br>CGGTAGACAAAATCATCCGCGCATGA	MTTQTFTLRDIAIAANA<br>KHGMDTTAAEDIARTY<br>LDQMDAEDGIERDEDEI<br>TQDDFDFLLGAIDSARR<br>AGDLGLHELDLISEAAL<br>EMQSQEDRLRAARDER<br>DAAIRAAVHAGARIQD<br>VATAAGISRQAVDKIIR<br>A
CSP1_59	-	38545	38204	*orf59*	hypothetical protein	ATGACCACCTACTACCGCATCCAGTCCCAGAACCGCCCCAACATCCTC<br>GACCCGGAAAACCAGTACTCCTACTCCTGGAACGACCTGGGCGCAGA<br>CCCACGCCACGGCATCAGCGTCATGGACGACCGCGAATCCCTCGCAG<br>AGTACATCGCCCAGACCGGCATCCAGTGGGACGAGACCTGGGAGCTG<br>CTCGAAGTCGAGGGCACCACCTCCGAGGATGAGGACGAGGACGCACA<br>CATGGGGGCTCGCCTCATCATCCCCACCGCCATCATCTCCCGCGAGCC<br>ACTCACCGACGGCTTCATGGAAGAAATCTTCGATGCCTTCGAGCAGCT<br>CGCAGCCTAA	MTTYYRIQSQNRPNILD<br>PENQYSYSWNDLGADP<br>RHGISVMDDRESLAEYI<br>AQTGIQWDETWELLEV<br>EGTTSEDEDEDAHMGA<br>RLIIPTAIISREPLTDGFM<br>EEIFDAFEQLAA
CSP1_60	-	39101	38670	*orf60*	hypothetical protein	ATGGATATTTACGAAACCTCATATGACGAATACGGCCCCGAATGGCA<br>CGCCACCCTACCCGATGATGTGCGCAAGCCAGTATTCCCCCACAATCC<br>AGCGCACGCCCCACGCAGTGGCCTAGCCCTACGCGTAGCCCGCGAAT<br>CATTAGGAATCAGTGCAGCATACCTAGCCGAATACCTAGGGGTAGGT<br>AAGCGCACGGCAGAGCGGTGGGAGAAGCTCGATGAAATCCCCGGGT<br>GGGTAGAAACTGCTCTCCGCAAAATCACCTTTGCTACCCAGGTGTGGG<br>AATCGGATTTACAGGATGCTGGGCAGGTCATGATCCACACCGGCGGC<br>TACCGCATGCTGGATGGTCGGCCACTACCTGAATCGTGGTGGATACAC<br>CTTGTGGGCCAGGCCATGCGCACTAATGCAACTATCATCCCAATCGCT<br>GACTAA	MDIYETSYDEYGPEWH<br>ATLPDDVRKPVFPHNP<br>AHAPRSGLALRVARES<br>LGISAAYLAEYLGVGK<br>RTAERWEKLDEIPGWV<br>ETALRKITFATQVWESD<br>LQDAGQVMIHTGGYR<br>MLDGRPLPESWWIHLV<br>GQAMRTNATIIPIAD

CSP1_61	-	39435	39145	*orf61*	hypothetical protein	ATGACCACCCTCACCTACTCCGCAGAGCTGGAACTCGCCAACAACGG AATCACCCACACCACCGCAACCATCGACACCTACGGCGACACCGCCG CAGAAGTCACCAAAGACATGCTCAACTTCTTCAACTCCCCCGACTATG AGGGCAGCCTGCAAGACTTCGAGGTCGTAACCCAGGAGGTCGACGAG GACTTTGCAGAGATCATCATCCAGGGCACCATCGCAGAAGCCGACGA GGACACCAACGAGGAAACCGAATACATCGCAACCGTCACCATCATTG GACGCTAA	MTTLTYSAELELANNGI THTTATIDTYGDTAAEV TKDMLNFFNSPDYEGS LQDFEVVTQEVDEDFA EIIIQGTIAEADEDTNEE TEYIATVTIIGR
CSP1_tRNA01	+	4492	4565	-	-	TCTCTGTTAGCTCAATTGGTAGAGCAGTGGACTTTTAATCCACGGGTT CCGGGTTCGAGTCCCGGGCGGAGAAC	

* https://toolkit.tuebingen.mpg.de/tools/hhpred

Table S2. Global sequence alignment of putative proteins (Orfs) of CSP1 with representative phage (MGEs, mobile genetic elements) presented in NCBI

Annotation		*Identity (%) & similarity (%) Compared with			
CSP1		*Corynebacterium striatum* strain 216 chromosome （CP024932.1 ）locus_tag		Corynebacterium phage IME1320_01 (KY653127.1) locus_tag	
tyrosine-type recombinase/integrase	Orf1	93.5% & 97.5%	A9D01_05720	23.6% & 37.8%	ARM68397.1
excalibur calcium-binding domain-containing protein	Orf2	97.9% & 98.3%	A9D01_05715	19.6% & 30.2%	ARM68398.1
hypothetical protein	Orf3	79.0% & 80.4%	A9D01_05710	38.9% & 49.3%	ARM68399.1
hypothetical protein	Orf4	15.9% & 29.9%	A9D01_05700		
hypothetical protein	Orf5	22.7% & 34.1%	A9D01_05695		
antA/AntB antirepressor family protein	Orf6	87.5% & 90.8%	A9D01_05690		
helix-turn-helix domain-containing protein	Orf7				
hypothetical protein	Orf8	93.2% & 97.3%	A9D01_05675		
		94.3% & 96.6%	A9D01_05670		
hypothetical protein	Orf10	97.0% & 98.5%	A9D01_05665		
YqaJ viral recombinase family protein	Orf11	95.4% & 97.7%	A9D01_05660		
hypothetical protein	Orf12	96.1% & 97.6%	A9D01_05655		
RecT-like ssDNA annealing protein	Orf13	93.9% & 96.2%	A9D01_05650		
single-stranded DNA-binding protein	Orf14	93.1% & 95.2%	A9D01_05645		
hypothetical protein	Orf15				
hypothetical protein	Orf16	95.6% & 96.7%	A9D01_05640		
hypothetical protein	Orf17	38.6% & 39.0%	A9D01_05635		

hypothetical protein	Orf18				
hypothetical protein	Orf19				
hypothetical protein	Orf20	80.1% & 82.9%	A9D01_05630		
hypothetical protein	Orf21				
hypothetical protein	Orf22	92.0% & 95.6%	A9D01_05625		
hypothetical protein	Orf23	34.9% & 40.1%	A9D01_05620		
hypothetical protein	Orf24				
hypothetical protein	Orf25				
hypothetical protein	Orf26				
hypothetical protein	Orf27				
hypothetical protein	Orf28				
hypothetical protein	Orf29				
RusA family crossover junction endodeoxyribonuclea se	Orf30	34.1 & 44.9	A9D01_05615		
hypothetical protein	Orf31	28.0% & 44.5%	A9D01_05610		
HNH endonuclease	Orf32	82.1% & 93.7%	A9D01_05605	84.2% & 94.7%	ARM68420.1
hypothetical protein	Orf33	91.0% & 94.6%	A9D01_05600	99.1% & 99.1%	ARM68421.1
terminase large subunit	Orf34	95.7% & 97.2%	A9D01_05595	96.2% & 97.6%	ARM68422.1
phage portal protein	Orf35	91.7% & 92.3%	A9D01_05590	85.3% & 85.6%	ARM68424.1
putative prohead protease	Orf36	98.9% & 98.9%	A9D01_05585	98.5% & 98.9%	ARM68425.1
major capsid protein	Orf37	98.8% & 98.8%	A9D01_05580	97.8% & 98.5%	ARM68426.1
hypothetical protein	Orf38	98.3% & 98.3%	A9D01_05575	96.7% & 96.7%	ARM68427.1
hypothetical protein	Orf39	98.4% & 98.4%	A9D01_05570	96.8% & 97.3%	ARM68428.1
hypothetical protein	Orf40	95.9% & 96.7%	A9D01_05565	96.7% & 97.5%	ARM68429.1
hypothetical protein	Orf41	97.8% & 97.8%	A9D01_05560	95.6% & 96.7%	ARM68430.1
hypothetical protein	Orf42	97.8% & 98.5%	A9D01_05555	96.3% & 97.8%	ARM68431.1
Ig domain-containing protein	Orf43	98.7% & 98.7%	A9D01_05550	90.2% & 92.1%	ARM68432.1
hypothetical protein	Orf44	99.2% & 99.2%	A9D01_05545	92.6% & 6.7%	ARM68433.1
Chromosome partition protein Smc/ Tail length tape-measure protein	Orf45	75.0% & 80.5%	A9D01_05535	66.8% & 76.6%	ARM68435.1
hypothetical protein	Orf46	98.9% & 98.9%	A9D01_05530	98.9% & 98.9%	ARM68436.1

putative tail protein	Orf47	61.5% & 62.1%	A9D01_05525	53.4% & 53.4%; 39.4% & 39.6%	ARM68437.1; ARM68438.1
hypothetical protein	Orf48	94.6% & 95.3%	A9D01_05520	98.4% & 98.4%	ARM68439.1
hypothetical protein	Orf49	98.4% & 98.8%	A9D01_05515	98.4% & 98.8%	ARM68440.1
hypothetical protein	Orf50	69.7% & 75.1%	A9D01_05510	69.9% & 75.8%	ARM68441.1
hypothetical protein	Orf51	65.5% & 70.2%	A9D01_05505	29.3% & 36.4%; 57.5% & 66.7%	ARM68442.1; ARM68443.1
hypothetical protein	Orf52	34.4% & 46.4%	A9D01_05500	77.0% & 86.9%	ARM68444.1
lysozyme M1 (1,4-beta-N-acetylmuramidase)	Orf53	77.2% & 84.8%	A9D01_05495	40.9% & 55.8%	ARM68445.1
putative membrane protein	Orf54	91.1% & 92.7%	A9D01_05490	56.5% & 66.1%	ARM68446.1
putative membrane protein	Orf55	95.0% & 97.9%	A9D01_05485	23.1% & 36.1%	ARM68447.1
putative membrane protein	Orf56	97.2% & 97.9%	A9D01_05480	17.5% & 31.3%	ARM68448.1
hypothetical protein	Orf57	92.7% & 95.4%	A9D01_05475		
hypothetical protein	Orf58	85.9% & 92.6%	A9D01_05470		
hypothetical protein	Orf59	88.6% & 95.6%	A9D01_05465		
hypothetical protein	Orf60				
hypothetical protein	Orf61				

*https://www.ebi.ac.uk/Tools/psa/emboss_needle/; This table only shows the information that the Identity or similarity of two protein sequences is ≧ 30%.

Table S3. Optimal MOI for phages

Phage (PFU/mL)	Bacteria (PFU/mL)	MOI(PFU/CFU)	CSP1 titer (PFU/mL)		
			**replicate 1**	**replicate 2**	**replicate 3**
10^5	10^8	0.001	5.28×10^9	4.88×10^9	5.22×10^9
10^6	10^8	0.01	4.80×10^9	4.72×10^9	4.76×10^9
10^7	10^8	0.1	6.37×10^{11}	4.84×10^{11}	4.77×10^{11}
10^8	10^8	1	4.00×10^{10}	4.39×10^{10}	5.68×10^{10}
10^9	10^8	10	4.50×10^9	6.27×10^9	3.20×10^9
10^{10}	10^8	100	5.40×10^9	6.24×10^9	5.60×10^9

Table S4. Data from the phage one-step growth curve assay

Time (min)	CSP1 titer (PFU/mL)			Time (min)	CSP1 titer (PFU/mL)		
	**replicate 1**	**replicate 2**	**replicate 3**		**replicate 1**	**replicate 2**	**replicate 3**
0	5.99×10^5	2.00×10^5	2.05×10^5	60	2.79×10^8	2.50×10^8	1.00×10^9
10	6.40×10^5	1.40×10^5	6.37×10^5	70	4.75×10^8	4.79×10^8	1.49×10^9
20	1.55×10^6	6.40×10^6	1.54×10^6	80	4.80×10^8	2.00×10^9	1.00×10^9
30	2.35×10^7	1.60×10^7	4.02×10^7	90	2.66×10^9	2.00×10^8	1.00×10^9
40	2.99×10^8	4.42×10^8	4.00×10^8	100	5.00×10^8	2.00×10^8	1.00×10^9
50	5.70×10^8	1.60×10^8	4.99×10^8				

Table S5. Evaluation of CSP1 phage cytotoxicity using the Cell Counting Kit-8 (CCK-8) assay

Incubation time (hour)	Phage (PFU)	HEK293 T cells viability (OD_{450nm})			A549 cells viability (OD_{450nm})		
		replicate 1	replicate 2	replicate 3	replicate 1	replicate 2	replicate 3
12	3×10^{10}	0.43085	0.41025	0.44645	0.51625	0.53225	0.52335
	3×10^{9}	0.39725	0.43065	0.42785	0.51835	0.50815	0.50245
	3×10^{8}	0.40365	0.40525	0.41655	0.59295	0.54445	0.49995
	3×10^{7}	0.42805	0.43555	0.40185	0.57895	0.50115	0.55465
	Control - 1	0.41325	0.43155	0.44405	0.54845	0.56135	0.56465
	Control - 2	0.41495	0.42055	0.40625	0.59665	0.53995	0.57765
24	3×10^{10}	1.35105	1.24525	1.33285	1.13125	1.05555	1.07335
	3×10^{9}	1.30495	1.39195	1.27885	1.24015	1.16155	1.08615
	3×10^{8}	1.39965	1.35755	1.34885	1.13765	1.18885	1.01975
	3×10^{7}	1.40945	1.31925	1.45365	1.09205	1.10515	1.02895
	Control - 1	1.45395	1.31045	1.43425	1.09015	1.18825	1.21325
	Control - 2	1.41405	1.39685	1.37755	1.24375	1.01095	1.01075

Table S6. Titers of phage CSP1 before and after incubation with cells in cytotoxicity assessment

Phage concentration		After incubated with HEK293 T cells Log10 (pfu/mL)		After incubated with A549 cells Log10 (pfu/mL)	
		12 h	24 h	12 h	24 h
Initial titer 3×10^{12} pfu/mL	Stock solution (10 µL) + 100 µL cells	11.53 ± 0.15	11.46 ± 0.03	11.18 ± 0.30	11.51 ± 0.56
	Stock solution diluted 10-fold (10 µL) + 100 µL cells	10.26 ± 0.04	10.29 ± 0.01	10.18 ± 0.10	10.39 ± 0.11
	Stock solution diluted 100-fold (10 µL) + 100 µL cells	9.42 ± 0.12	9.54 ± 0.06	9.41 ± 0.24	9.59 ± 0.11

	Stock solution diluted 1000-fold (10 μL) + 100 μL cells	8.37 ± 0.07	8.23 ± 0.25	8.40 ± 0.14	8.10 ± 0.35
